# Deliberations on the External Morphology and Modification of the Labial Segments in the Nepomorpha (Heteroptera: Insecta) with Notes on the Phylogenetic Characteristics

**DOI:** 10.1155/2013/790343

**Published:** 2013-10-31

**Authors:** Jolanta Brożek

**Affiliations:** University of Silesia, Faculty of Biology and Environmental Protection, Department of Zoology, Bankowa Street 9, 40-007 Katowice, Poland

## Abstract

The present study provides new data concerning the external morphology of the labial segments of 46 species from nine Nepomorpha families using the scanning electron microscope. The labial segments are described in detail and images of their structures are presented for the systematic groups. Subsequent segments of the labium (I, II, III, and IV) are shaped similarly in all investigated taxa but carry individual characters in some (sub-)families. Five morphologically distinct forms of the apical plate and five intercalary sclerites have been identified. Additionally, three types of the articulation on the dorsal side between the third and second segments are interpreted as the new characters. The presence of the midventral condyle on the distal edge of the first segment and the third segment has been reanalyzed. New position of the midventral condyle on the proximal edge of the fourth labial segment has been distinguished in several groups. The new set of characters has been estimated from the plesiomorphic taxa of the Nepoidea (Nepidae and Belostomatidae) and subsequently through the more advanced taxa in the relation to the outgroup (Gerromorpha). The evaluation of these characters has revealed twenty-seven new apomorphies for the labium in the Nepomorpha.

## 1. Introduction

Hemiptera is one of the numerous insect orders comprising more than 40,000 described species [1]. The extraordinary diversity in terms of morphology and lifestyle adjustments has long attracted the attention of evolutionary biologists and systematicians. The hemipterans have been classified into four major taxa (suborder): Heteroptera, Coleorrhyncha, Sternorrhyncha, and Auchenorrhyncha [2]; the latter are divided into Fulgoromorpha and Cicadomorpha [3]. The suborder Heteroptera contains seven infraorder [2, 4] in it Nepomorpha or eight, with regard to the Aradimorpha [5]. The water bugs belonging to the Nepomorpha infraorder include taxa previously placed either in the group of the Hydrocorisae [6–10] or in the group of the Cryptocerata [8, 11, 12]. Presently, the Nepomorpha include thirteen families [13, 14]: eleven aquatic families and two littoral families (the Gelastocoridae and Ochteridae).

Differences in the forms of the insects mouthparts are related to their diets, but two basic types can be recognized: one adjusted to biting and chewing solid food and the other adjusted to sucking up fluids [15]. These basic types of insects mouthparts occur with numerous modifications of their structural elements and reflect the variety of food available, peculiar to various groups of insects. The Hemiptera are characterized by a strong modification of their feeding apparatus into a rostrum consisting of the labium guiding two pairs of respective mandibular and maxillary stylets enabling the penetration into nutritional tissues of plants or animals, or both (mixed) [15, 16]. The elements of this apparatus together with the feeding and salivary pumps placed in the hypopharynx form the piercing and sucking type of apparatus [17]. However, there are deviations from the general model of the hemipteran mouthparts found in the representatives of the Gerromorpha and Nepomorpha infraorders. According to Cobben [18], modes of feeding depended on the external structure of the maxillae and mandibles. One of them, the scratching-filtration mode, is typical for bugs with strongly dentate and bristly maxillary stylets. The morphological study has shown that the mandibular and maxillary stylets are suitable for the scratching-filtration mode; however, a clear trend for their modification can be observed in particular taxonomic groups of the Nepomorpha [19]. 

The labial structure is generally stable and similar in most families of the Nepomorpha except for the Corixidae [9, 20, 21]. The latter differ considerably from the remaining nepomorpha families: the labium of the corixids is shorter and broader, without a distinct fourt-part segmentation [20–23], (Brożek, submitted, 2013). Several possible interpretations of its homologies with the labium of the typical Nepomorpha have been discussed in the papers of the previously mentioned authors.

In most Nepomorpha as well as in most other heteropterans (e.g., Leptopodomorpha, Gerromorpha, and Pentatomomorpha), the labium is typically four-segmented [4, 9, 18, 20]. The Reduviidae (Cimicomorpha) is an exception, with a three-segmented structure of the labium resulting from the reduction of the first segment [24]. The labium of heteropterans is usually straight or slightly curving, generally representing a tubular shape of varied length. The short labium reaching slightly towards the posterior margin of the head is typical for most Nepomorpha, except for the Ochteridae and Aphelocheiridae. In the latter mentioned families, as well as in the Reduviidae (Cimicomorpha), the labium is long, reaching near the apex of the abdomen similarly as in some Pentatomomorpha [4, 20, 24, 25].

The morphological and anatomical structures of the labium in the individual representatives of the Nepomorpha have been subject of observation and interpretation for a number of years [9, 21, 26–29]. In dozens of species, the labial structures have been analyzed by Mahner [12], especially in the context of the phylogenetic meaning. The comprehensive descriptions and comparisons of the characters of the labium in several representatives of seven families of the Nepomorpha have been presented by Parsons [20, 25]. According to data provided by this author, the labium in typical Nepomorpha (= Hydrocorisae) posseses several characteristic external structural elements for particular families. Generally, the first segment is often reduced while the third segment is typically the longest. A median stylet groove on the dorsal surface of the labium contains the stylet bundle and this groove is sclerotized in the first segment; elsewhere, it is predominantly membranous but reinforced by localized sclerotized regions. In most nepomorpha, in the two terminal segments and in at least the distal half of the second segment, the lips (margins) of the stylet groove touch each other, forming a closed canal. However, proximally, the groove is usually open dorsally and the stylet bundle is held in place by the lobe-like terminal portions of the labrum and epipharynx, which overlap the base of the labium. In the distal part of the third segment, the stylet groove contains a muscle-controlled, sclerotized holdfast mechanism which, by varying the diameter of the groove, regulates the movement of the enclosed stylets. In some Nepomorpha, a pair of small intercalary sclerites is connected with this device and lies middorsally between the third and fourth segments. The joints between the first and second and the third and fourth segments are monocondylic, with a single midventral point of articulation; movement at these joints is primarily rotary. The second and third segments are joined by a dicondylic articulation, the two points of contact lying dorsolaterally. A similar dicondylic joint also connects the first segment to the head.

The last segment apically is tripartite and consists of two lateral lobes, which are equipped with the sensory structures and the ventral one, that is, the apical plate [18].

The present study focuses on describing the shape of intercalary sclerites and the apical plate and concerns also the shape of the segments, believing that these segments have undergone the greatest evolutionary changes in relation to the type of food in the taxonomic groups. These changes have been preserved as various modifications and forms of particular structures of the labium and would provide an interesting source for comprehensive studies. The evidence of such evolutionary changes may be the clear difference in the structure of the labium between the Corixoidea (short and triangular, almost unsegmented labium) and the remaining taxa of the Nepomorpha (tubular, segmented labium of varying lengths, with several characteristic elements in particular families or species). Moreover, the external structures of the maxillae in the Corixoidea are clearly different from the corresponding structures in the other Nepomorpha [19, 21, 22]. These obvious differences are undoubtedly connected with types of nutrition. Members of the Corixoidea are characterized by various diets, as there are algivorous, detritophagous, and omnivorous species among them (some are also predators), while the rest of the Nepomorpha are mainly predators [9, 18]. These several factors encourage undertaking comprehensive studies of the labium. Moreover, most of the previously available information on the labium has been given in the form referring to just a single taxon or several taxa. A systematic comprehensive evaluation of the data in evolutionary context is still to be made. The latest studies, a combined morphological and molecular analysis [30] and the analysis of the relationship of families based on the mitochondrial genome in nine nepomorphan taxa [31], have shown distinct discrepancies of the model status in the systematics of the Nepomorpha and the relationships between families. Therefore, it has been decided to undertake a morphological study of the labium in the systematic groups of the Nepomorpha in order to learn about their diversity and also their importance for phylogenetic reasoning.

In the present study, 46 species of the nepomorphans were examined by means of scanning electron microscopy and results were compared mainly with the external labial morphology described in several species by Quadri [21], Parsons [20, 25], and Mahner [12]. 

The aims of the study were to: (1) provide new descriptions of morphological characters of the labium, (2) represent their properties, respectively, in the systematic groups, and (3) indicate new sets of characters that could potentially be used in the future for the cladistic analysis of the Nepomorpha.

## 2. Materials and Methods

### 2.1. Taxon Samples

This study of the labium was based on dry material consisting of adult specimens from the collections of the Natural History Museum in Vienna, Zoological Museum of the State Moscow University and the Paleontological Institute of the Russian Academy of Sciences in Moscow. The basal part of the head with a part of the rostrum or the whole specimens was glued onto a scanning electron microscope stub. The labial segments used for SEM photographs were not coated; the photographs were taken with a Hitachi scanning electron microscope, with the samples placed in the low-pressure chamber.

Classification and order of families and subfamilies listed in Table 1 are the same as those established by Štys and Jansson [32], except for the Micronectidae and the Diaprepocoridae which have been elevated to the rank of family [13, 14]. 

In the Discussion section, morphology of the labial segments and the preliminary estimation of the characters of the labium with respect to their phylogenetic value are compared with the basic model within the group (i.e., the basal taxa of the Nepidae and Belostomatidae) and to the more diverse forms of these structures in more evolutionarily advanced groups (i.e., Ochteridae, Gelastocoridae, Aphelocheiridae, Naucoridae, Pleidae, Helotrephidae, and Notonectidae, resp.) in relation to the characters of gerromorphans described by Andersen [33] and treated as an outgroup for the Nepomorpha by Wheeler et al. [34]. The model of the labium in representatives of the Corixidae, Diaprepocoridae, and Micronectidae has been described elsewhere (Brożek, submitted, 2013). 

SEM documentations have been posted in Figures 1–5 in Section 3 and in Appendix (see Figures 6–33).

### 2.2. Researched Taxa: Previously Gathered Data (Table 2)

For the sake of conducting a comprehensive comparison of previous and current studies, Parsons's [20, 25] data are given here, referring to the external characters of the labium. Additionally, Parson's results have been supplemented with Mahner's data [12].

## 3. Results

### 3.1. Various Shapes of the Apical Ventral Plate of the Labium in the Nepomorphan Taxa

**Figure 1:**
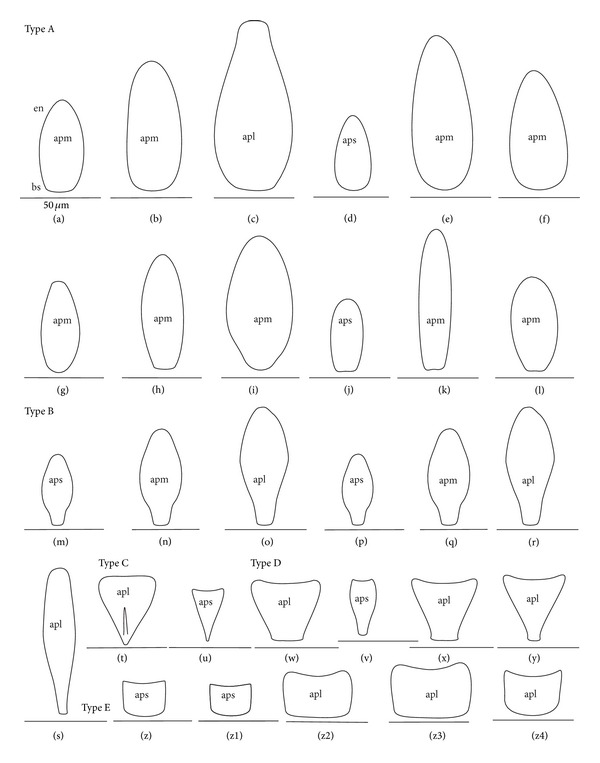
Various shapes of the apical plate in the Nepomorpha: Nepinae: (a) *Curicta granulosa*, (b) *Borborophyes mayri*, (c) *Laccotrephes japonensis*, (d) *Nepa cinerea*, Ranatrinae: (e) *Cercotmetus asiaticus*, (f) *Ranatra chinensis*, Ochteridae; (g) *Ochterus marginatus*, Aphelocheiridae: (h) *Aphelocheirus aestivalis*, Naucoridae: Limnocorinae: (i) *Limnocoris lutzi*, Helotrephidae: (j) *Tiphotrephes indicus*, (k) *Hydrotrephes visayasensis*, (l) *Hydrotrephes balnearius*, Belostomatidae: Belostomatinae: (m) *Belostoma flumineum*, (n) *Deinostoma dilatatum*, (o) *Appasus major*, (p) *Hydrocyrius colombiae*, (q) *Limnogeton fieberi*, Lethocerinae: (r) *Lethocerus deyrollei*, Gelastocoridae: Nerthrinae: (s) *Nerthra nepaeformis*, Gelastocorinae: (t) *Gelastocoris oculatus*, Pleidae: (u) *Paraplea frontalis*, Notonectidae: Anisopinae: (w) *Anisops camaroonensis*, Notonectinae: (v) *Nychia sappho*, (x) *Enithares bergrothi*, (y) *Notonecta glauca*. Naucoridae: Cheirochelinae: (z) *Cheirochela feana*, (z1). *Coptocatus oblongulus*, Cryphocricinae: (z2) *Cryphocricos hungerfordi*. Laccocorinae: (z3) *Laccocoris hoogstraali*, Naucorinae: (z4) *Naucoris maculatus*. The same scale bar has been applied for all images (50 *μ*m), bs: base of the apical plate, en: tip of the apical plate.

The apical plate is situated ventrally at the end of the fourth labial segment below the exit of the stylets bundle (maxillae and mandibles). The base (proximal part) of the apical plate emerges from the ventral side of the labium; the end (distal part) and lateral side of the apical plate are free. The apical plate of the labium of the investigated species is characterized by diversified forms and sizes and therefore has been classified into five morphological types (A–E), listed later. The same scale bar has been applied to show the sizes of the apical plate; thus, the visible differences in size are characteristic for the particular species. In the present analysis, the sizes of the apical plate arbitrarily have been categorized into small (aps), medium (apm), and large (apl) (Figure 1) separately for the particular shape (Type A–E).

**Figure 2:**
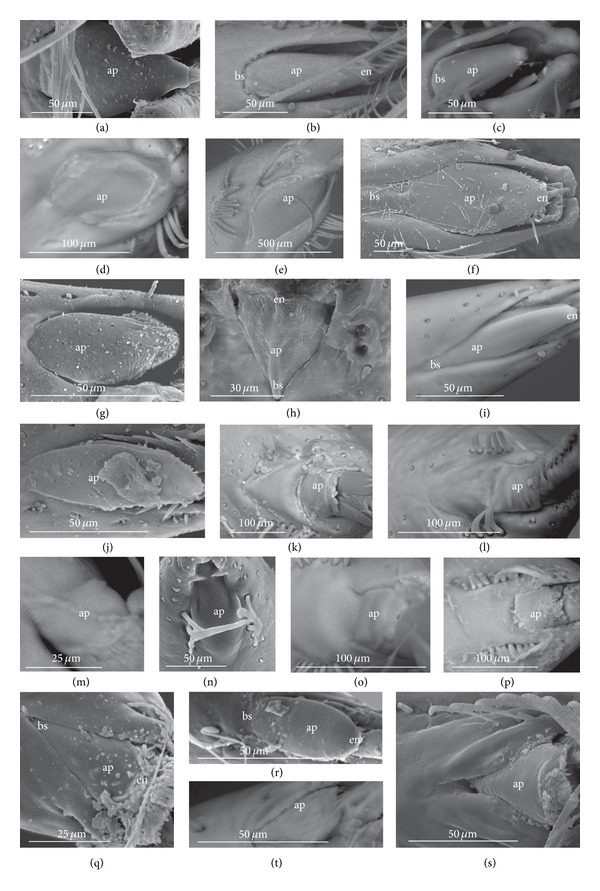
Various shapes of the apical plate in the Nepomorpha: Nepinae: (a) *Laccotrephes japonensis*, Ranatrinae: (b) *Cercotmetus asiaticus*, (c) *Ranatra chinensis*, Belostomatidae: Belostomatinae: (d) *Belostoma flumineum*, (e) *Hydrocyrius colombiae*, Lethocerinae: (f) *Lethocerus deyrollei*, Ochteridae: (g) *Ochterus marginatus*, Gelastocoridae: Gelastocorinae: (h) *Gelastocoris oculatus*, Nerthrinae: (i) *Nerthra nepaeformis*, Aphelocheiridae: (j) *Aphelocheirus aestivalis*, Naucoridae: Cheirochelinae: (k) *Cheirochela feana*, Cryphocricinae: (l) *Cryphocricos hungerfordi*. Laccocorinae: (m) *Laccocoris hoogstraali*, Limnocorinae: (n) *Limnocoris lutzi*, Naucorinae: (o) *Pelocoris femoratus*, (p) *Naucoris maculatus*, Pleidae: (q) *Paraplea frontalis*, Helotrephidae: (r) *Hydrotrephes visayasensis*, Notonectidae: Notonectinae: (s) *Notonecta glauca*, (t) *Nychia sappho*.

*Type A*. The apical plate (ap) is generally oval-shaped (longer than wider) with slightly modified forms and sizes (small, medium, and large) in different species as is visible in the Figures 1(a)–1(l). The base (bs) of the plate is slightly wider than its end (en). The oval apical plate has been found in representatives of two subfamilies of the Nepidae (Nepinae: *Curicta granulosa* (Figure 1(a); apm), *Borborohyes mayri* (Figure 1(b), apm), *Laccotrephes japonensis* (Figure 1(c), apl, Figure 2(a)), *Nepa cinerea* (Figure 1(d), aps) and Ranatrinae: *Cercotmetus asiaticus* (Figure 1(e), apm Figure 2(b)), *Ranatra chinensis* (Figure 1(f), apm Figure 2(c)), as well as in the Ochteridae (*Ochterus marginatus* (Figure 1(g), apm Figure 2(g)), Aphelocheiridae (*Aphelocheirus aestivalis *(Figure 1(h), apm Figure 2(j) *A. variegatus*), Naucorinae (Limnocorinae: *Limnocoris lutzi* (Figure 1(i), apm Figure 2(n)) and Helotrephidae (*Tiphotrephes indicus*, *Hydrotrephes visayasensis*, *H. balnearius*, Figure 1(j) aps, Figures 1(k) and 1(l), apm, Figure 2(r)).

*Type B*. The apical plate (ap) is palm-shaped and is characterized by a narrow base, wider medially and slightly narrowing at the end. This type of different sizes (aps and apm) has been observed in various species representing the Belostomatinae; *Belostoma flumineum* (Figure 1(m), aps Figure 2(d)), *Deinostoma dilatatum *(Figure 1(n), apm), *Appasus major* (Figure 1(o), apl), *Hydrocyrius colombiae* (Figure 1(p), aps Figure 2(e)), *Limnogeton fieberi *(Figure 1(q), apm), and Lethocerinae *Lethocerus deyrollei *(Figure 1(r), apl, Figure 2(f)). In addition, in the Nerthrinae (*Nerthra nepaeformis* (Figure 1(s), apl Figure 2(j)) and *N. marcothorax,* the apical plate is slimmer (the shape of a slim palm) than that in the belostomatidae species. 

*Type C*. The apical plate (ap) is approximately triangular-shaped with the pointed base and wider at the end. This shape of the plate has been found in the Gelastocorinae (*Gelastocoris oculatus* Figures 1(t) and 2(h)) and Pleidae (*Paraplea frontalis* Figures 1(u) and 2(q)). Two sizes of the apical plate have been classified as large (apl) in *Gelastocoris* and small in *Plea *(aps). 

*Type D*. The apical plate (ap) is trapezoid-shaped. The distal margin is wider than the base margin. In several species, the distal margin is slightly concave. This type has been characteristic for the Notonectidae: Anisopinae (*Anisops camaroonensis* Figure 1(w)) and the Notonectinae (*Nychia sappho* Figures 1(v) and 2(t); *Enithares bergrothi* Figures 1(x) and 32(c), *Notonecta glauca* Figures 1(y) and 2(s)). Among those species (Figures 1(w), 1(x), and 1(y), apl), the apical plate is smaller in *Nychia *(Figure 1(v), aps). 

*Type E*. The apical plate (ap) is rectangle-shaped. This plate is wider than longer and has a slightly concave distal margin. This type of plate can be observed in the Naucoridae: *Cheirochela feana* (Figures 1(z) and 2(k)); *Gestroiella limnocoroides*, *Tanycricos longiceps *(Figure 21(f)), *Coptocatus kinabalu*, *Coptocatus oblongulus* (Figure 1(z1)); *Ambrysus occidentalis*, *Cryphocircos hungerfordi* (Figures 1(z2) and 2(l)); *Laccocoris hoogstraali* (Figures 1(z3) and 2(m)); *Naucoris maculatus* (Figures 1(z4), 2(p) and 2(l)); *Pelocoris femoratus* (Figure 2(o)), *Ilyocoris cimicoides* (Figure 25(b)), *Namtokocoris siamensis*, *Macrocoris rhantoides* (Figure 26(b)), *Neomacrocoris handlirschi* (Figure 26(e)) except for the Limocorinae. Among those species, the apical plate is smaller (aps) in the species representing the Cheirochelinae (Figures 1(z) and 1(z1)) than in the remaining species (Figures 1(z2), 1(z3) and 1(z4)).

### 3.2. Various Shapes of Intercalary Sclerites of the Labium in Several Taxa

A pair of intercalary sclerites (is) lie dorsally in the distal part of the third labial segment and partly overlap the proximal part of the fourth segment of the labium. These structures are connected with the internal mechanism (holdfast device). In the basal part, the sclerites are equipped with the membrane (ms) (intersegmental membrane according to Parsons [20]), so that they can budge. In several taxa, this membrane is reduced and the movement of plates is limited. In this study, four different forms of intercalary sclerites have been identified as follows.

**Figure 3:**
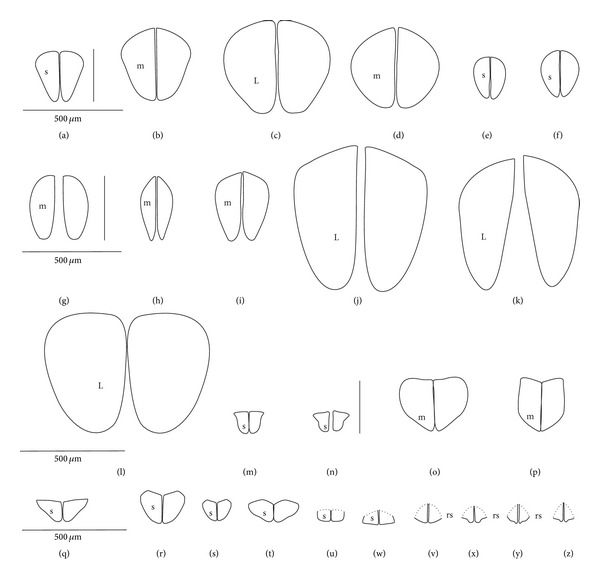
Various shapes of intercalary sclerites in the Nepomorpha: Nepinae: (a) *Curicta granulosa*, (b) *Borborophyes mayri*, (c) *Laccotrephes japonensis*, (d) *Nepa cinerea*, Ranatrinae: (e) *Cercotmetus asiaticus*, (f) *Ranatra chinensis*. Belostomatidae: Belostomatinae: (g) *Belostoma flumineum*, (h) *Deinostoma dilatatum*, (i) *Appasus major*, (j) *Hydrocyrius colombiae*, (k) *Limnogeton fieberi*, Lethocerinae: (l) *Lethocerus deyrollei*, Ochteridae: (m) *Ochterus marginatus*, Gelastocoridae: Gelastocorinae: (n) *Gelastocoris oculatus*, Nerthrinae: (o) *Nerthra nepaeformis*, Aphelocheiridae: (p) *Aphelocheirus aestivalis*, Naucoridae: Cheirochelinae: (q) *Cheirochela feana*, (r) *Gestroiella limnocoroides*, (s) *Coptocatus oblongulus*, (t) *Tanycricos longiceps*, Limnocorinae: (u) *Limnocoris lutzi*, Cryphocricinae: (w) *Cryphocricos hungerfordi*, Laccocorinae: (v) *Laccocoris hoogstraali*, Naucorinae, (x) *Naucoris maculatus*, Notonectidae: Anisopinae: (y) *Anisops camaroonensis*, Notonectinae: (z) *Notonecta glauca*. The dotted line indicates a border between the third labial segment and the intercalary sclerites. The same scale bar has been applied for all images (500 *μ*m).

**Figure 4:**
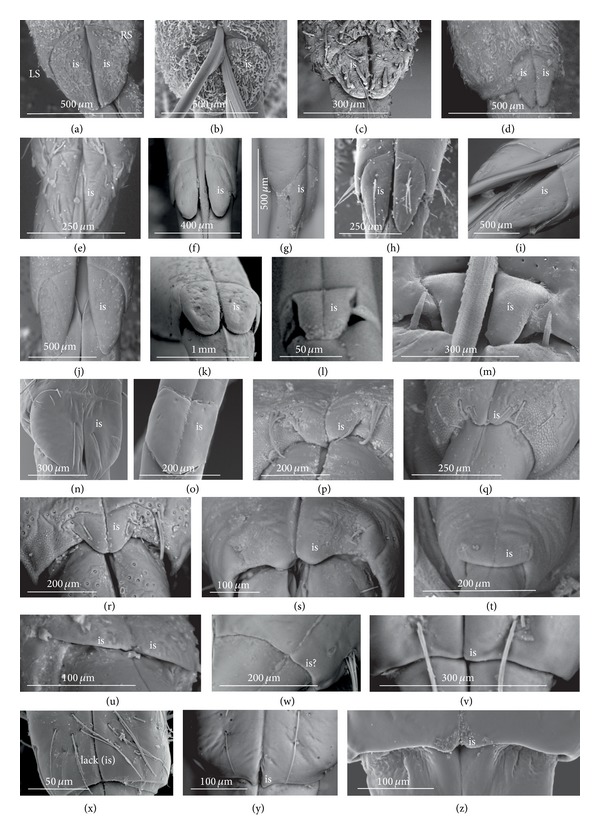
Examples of diversified intercalary sclerites in the Nepomorpha. Nepinae: (a) *Borborophyes mayri*, (b) *Laccotrephes japonensis*, (c) *Nepa cinerea*, Ranatrinae: (d) *Cercotmetus asiaticus*, (e) *Ranatra chinensis*. Belostomatidae: Belostomatinae: (f) *Belostoma flumineum*, (g) *Deinostoma dilatatum*, (h) *Appasus major*, (i) *Hydrocyrius colombiae*, (j) *Limnogeton fieberi*, Lethocerinae: (k) *Lethocerus deyrollei*, Ochteridae; (l) *Ochterus marginatus*, Gelastocoridae: Gelastocorinae: (m) *Gelastocoris oculatus*, Nerthrinae: (n) *Nerthra nepaeformis*, Aphelocheiridae: (o) *Aphelocheirus aestivalis*, Naucoridae: Cheirochelinae: (p) *Cheirochela feana*, (q) *Gestroiella limnocoroides*, (r) *Coptocatus oblongulus*, (s) *Tanycricos longiceps*, Limnocorinae: (t) *Limnocoris lutzi*, Cryphocricinae: (u) *Cryphocricos hungerfordi*, Laccocorinae: (w) *Laccocoris hoogstraali*, Naucorinae: (v) *Naucoris maculatus*, Pleidae: (x) *Paraplea frontalis*, Notonectidae: Anisopinae: (y) *Anisops camaroonensis*, Notonectinae: (z) *Notonecta glauca*.

*Type A*. The pair of plates (flaps) is placed dorsally but does not reach the lateral side of the labial segment (Figures 3(a)–3(l) and 4(a)–4(k)). Three different sizes of flaps, classified as small (s), medium (m), and large (L), have been distinguished (Figures 3(a)–3(l)) in the investigated species of belostomatids and nepids.

*Type B*. The small flaps (narrow and short) are evidently situated in the middle of the dorsal side of the third labial segment. The form of intercalary sclerites is typical for the Ochteridae (Figures 3(m) and 4(l)) and Gelastocorinae (Gelastocoridae) (Figures 3(n) and 4(m)).

*Type C*. The medium size flaps are subtriangular-shaped and overlap the lateral side of the labial segment. However, in the *Nerthra* (Figure 3(o)), these sclerites are wider than in the *Aphelocheirus *(Figure 3(p)). This shape occurs in the Nethrinae (Figures 3(o) and 4(n)) (Gelastocoridae) and Aphelocheiridae (Figures 3(p) and 4(o)). 

*Type D*. This type has been found in some of the Naucoridae. In the Cheirochelinae (Figures 3(q)–3(t) and 4(p)–4(s)), Limnocorinae (Figures 3(u) and 4(t)), and Cryphocricinae (Figures 3(w) and 4(u)), the intercalary sclerites are small flaps with a slightly distinct membrane at the base. 

*Type E*. The flaps are severely reduced, the membrane is not visible, and only traces of the sclerites are present in the form of a small extension on the distal edge of the third segment. This type has been observed in the Laccocorinae (Figures 3(v) and 4(w)) and Naucorinae (Figures 3(x) and 4(v)). A similar structure can be noticed in the Notonectidae (*Anisops camaroonensis* (Figures 3(y) and 4(y)), *Nychia sappho*, *Enithares bergrothi,* and *Notonecta glauca *(Figures 3(z) and 4(z)). 

The distinct lack of intercalary sclerites and also lack of their traces can be observed in the Pleidae (Figure 4(x)) and Helotrephidae (Figures 29(a), 29(b), and 29(c)).

### 3.3. External Structures of the Labial Segments in the Systematic Groups of the Nepomorpha

**Figure 5:**
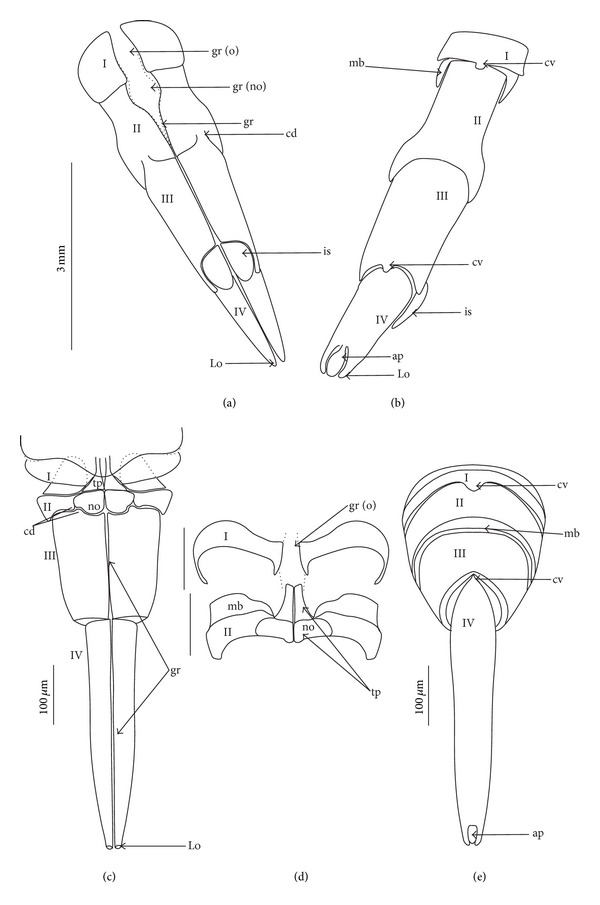
General model of the labium focusing on the character of the segment in the Belostomatidae and Helotrephidae. (a) dorsal side of the labial segments in *Hydrocyrius colombiae *(Belostomatidae), (b) ventral side of the labial segments in *Hydrocyrius colombiae *(Belostomatidae), (c) dorsal side of the labial segment in *Hydrotrephes balnearius* (Helotrephidae), (d) detailed shape of the I and II segments, dorsal view, (e) ventral side of the labial segments. ap: apical plate, cd: dorsal condyle, cv: midventral condyle, gr (o): open stylet groove, gr: closed stylet groove, gr (no): stylet groove has no definite lips, is: intercalary sclerites, lo: lateral lobe of apex, mb: intersegmental membrane, no: nodule of triangular plate, tp: triangular plate, I, II, III, IV numbers of the labial segments.

The modifications of the shape of the first and second labial segments on the dorsal side are more visible than on the ventral side. Generally, the shape of the ventral side of those segments is similar in all presently studied nepomorphan species. The dorsal surface of the first segment is usually uniform and ring-shaped, but there is a slight variation with respect to the size of this segment in particular groups. The type of the stylet groove has evident influence on the type of the dorsal structures of the first segment. When, dorsally, the edges (right and left) of the first segment and partially the edges of the second segment are not in contact, an open groove for the stylets is formed (Figure 5(a)). A reverse situation can also be observed: when, dorsally, the edges (right and left) of the first segment and the edges of the second segment are in contact, a closed groove for the stylets is formed (Figure 5(c)). From the ventral side, the first segment looks like a band (stripe), which is narrow or wide in different species. However, one of characteristic features of the first segment is the presence of the midventral condyle (cv) on the distal edge (Figures 5(b) and 5(e)), observed in most of the studied the species.

The dorsal surface of the second segment is uniform or divided (Figures 5(a), 5(c), and 5(d)), and the ventral side is either narrower or broader than the strip. Between these segments, there is a more or less developed membrane. However, the two first labial segments vary in the dorsal part from one family to another, as presented later. The connection between the second and third segments on the dorsal side is realized through the condyles and a membrane.

The third segment has a tubular shape and the fourth segment is conical; this pattern has been observed in all investigated species. A singular midventral condyle has been observed in several taxa on the distal edge of the third segment (Figure 5(b)) or on the proximal edge of the fourth segment (Figure 5(e)). Geometrical association of the shape for those segments has been used for a comprehensive analysis of the shape of the labial segments visible in the SEM. Only the external, sclerotized structures have been described in the way given in the following.

The intersegmental slerites (si) are marked on several images; they have not always been visible in comparison to the description provided by Parsons [20], and for this reason these structures are not discussed.

### 3.4. External Variation of the First Segment

**Figure 6:**
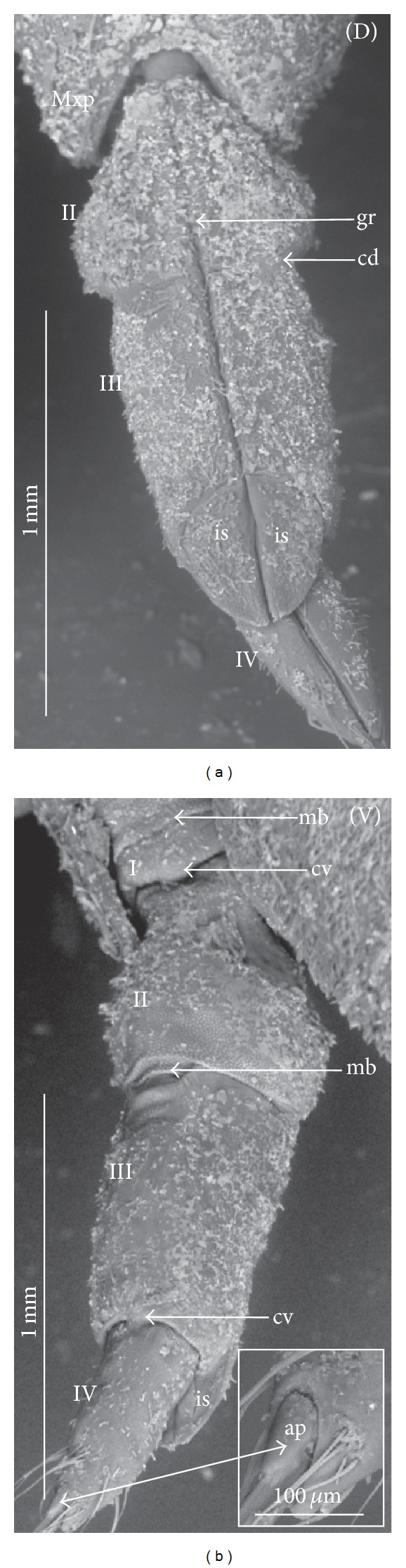
Shapes of the labial segments of the Nepidae (Nepinae). (a)-(b). *Borborophyes mayri*. (a) Dorsal view (D) of the labium, shapes of the II, III, and IV segments are visible, the stylet groove (gr) is opened, the articulation of the dorsal condyle (cd) between the II and III segments on the dorsal side, intercalary sclerites (is) on the III segment. (b) The shape of the first segment is visible, the base of the second (II) segment in narrower than the base of the first (I) segment, the midventral condyles (cv) on the distal edge of the first segment (I) and third segments are present, the oval apical plate and the intersegmental membranes are present. Mxp: maxillary plates.

**Figure 7:**
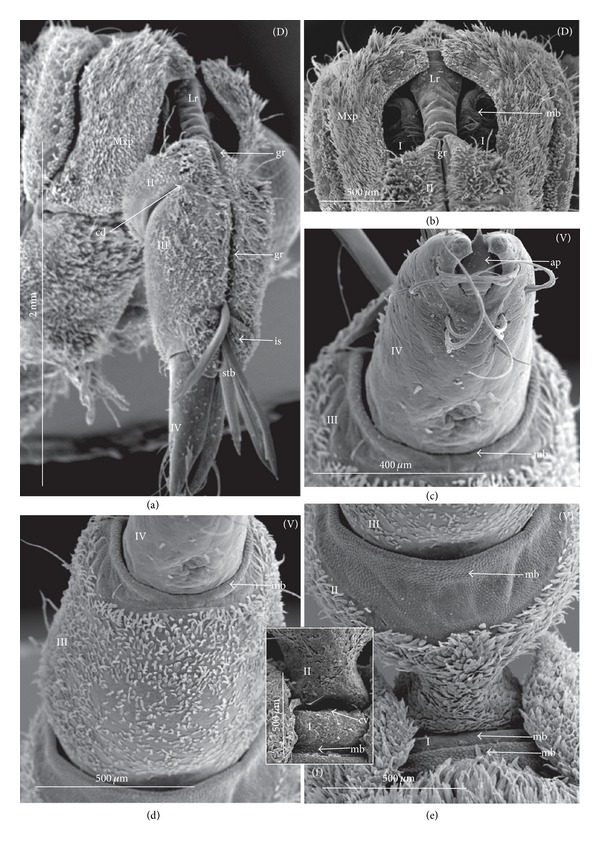
Shapes of the labial segments of the Nepidae (Nepinae). (a)–(e) *Laccotrephes japonensis*. (a) Dorsal view (D) of the labium, the II, III and IV segments are visible, the articulation of the dorsal condyle (cd) between the II and III segments on the dorsal side, intercalary sclerites (is) on the III, segment. (b) The shape of the first segment slightly visible in the dorsal position, the base of the second (II) segment is almost as wide as the end of the first (I) segment, the stylet groove (gr) is opened, (c) ventral view of the IV segment, the oval apical plate is visible, (d) ventral view (V), the connection between the third and fourth segments on the ventral side, the midventral condyle (cv) on the third segment is slightly visible, (e) the ventral view of the first and second segments (cuplike), the intersegmental membranes can be observed, (f) the midventral condyle (cv) on the distal edge of the first segment (I). Lr: labrum, Mxp: maxillary plates.

**Figure 8:**
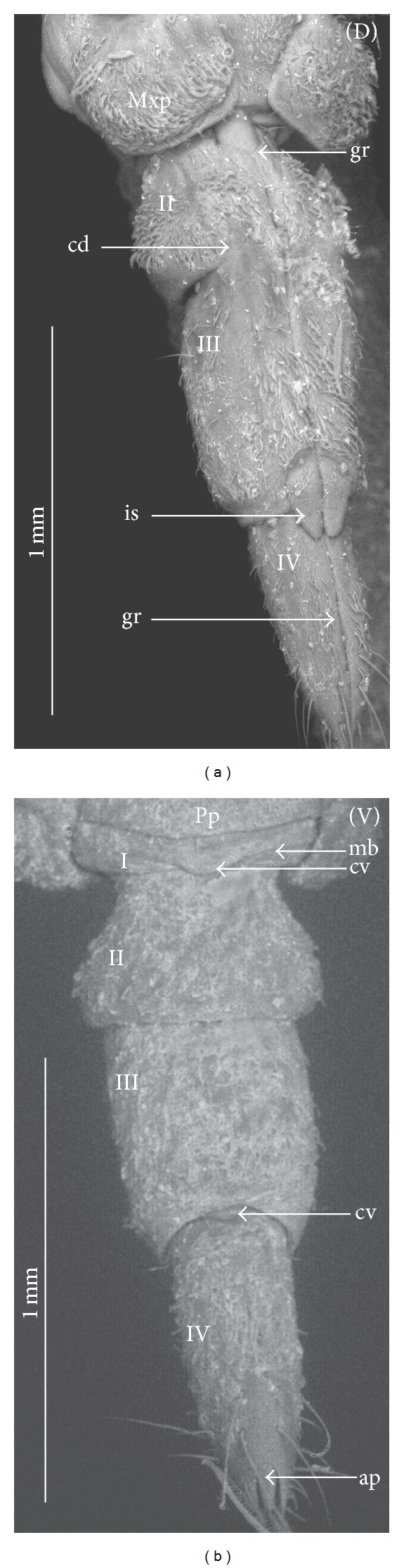
Shapes of the labial segments of the Nepidae (Ranatrinae). (a)-(b) *Cercotmetus asiaticus*. (a) Dorsal view (D) of the labium, the II, III and IV segments are visible, the articulation of the dorsal condyle (cd) between the II and III segments on the dorsal side, intercalary sclerites (is) on the III segment, the stylet groove (gr) is partly opened at the base of the second segment, only the remaining segment of the stylet groove is closed. (b) Ventral view (V), shape of the first segment is slightly visible with the midventral condyle (cv), the base of the second (II) segment in narrower than the end of the first (I) segment, the distal margin is wide, the connection between the third and fourth segment with the midventral condyle (cv) on the distal edge of the third segment, on the IV segment the oval apical plate is visible. Mxp: maxillary plates.

**Figure 9:**
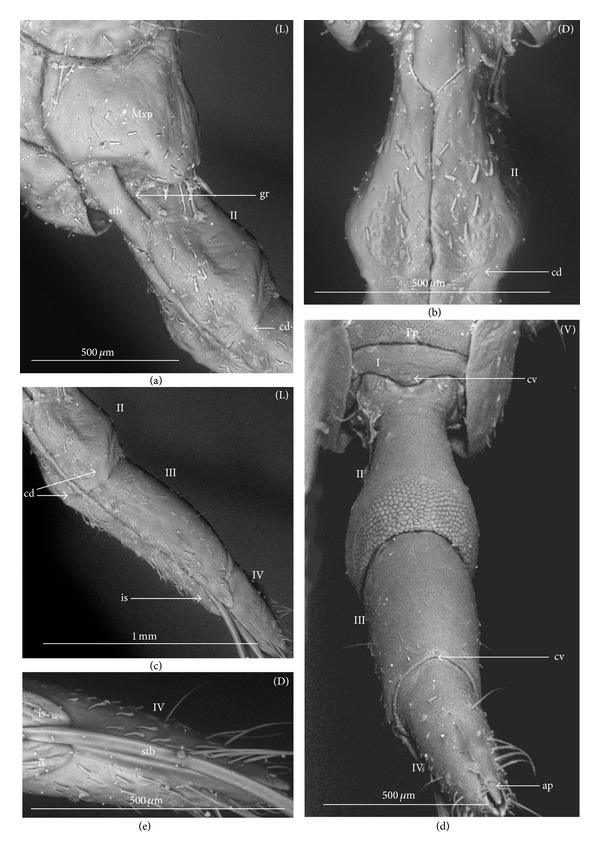
Shapes of the labial segments of the Nepidae (Ranatrinae). (a)–(e) *Ranatra chinensis*. (a) Lateral view (L) of the II segment, the stylet groove (gr) is partly opened at the base of the second segment, (b) the shape of the second segment, dorsal view (narrow the proximal part and a wider distal edge), the articulation of the dorsal condyle (cd) between the II and III segments on the dorsal side, (c) intercalary sclerites (is) on the III segment, the stylet groove is closed on the distal part of the second segment (II) and in the remaining segments (II and IV), (d) ventral view (V), the shape of the first segment with the midventral condyle (cv), the base of the second (II) segment in narrower than the end of the first (I) segment, the distal margin is wide, the connection between the third and fourth segments with the midventral condyle (cv) on the distal edge of the third segment, on the IV segment the oval apical plate is visible, and (e) the shape of the IV segment. Mxp: maxillary plates, Pp: posterior plate of the cranium, stb: stylets bundle.

*Nepidae.* Nepinae: *Curicta granulosa, Borborophyes mayri* (Figure 6), *Laccotrephes japonensis* (Figures 7(a)–7(e)), *Nepa cinerea*; Ranatrinae: *Cercotmetus asiaticus* (Figures 8(a), and 8(b)), *Ranatra chinensis* (Figures 9(a)–9(e)).

The segment from the dorsal side is a narrow stripe and, furthermore, only slightly visible in the investigated species of the Nepinae (Figures 7(a) and 7(b),* Laccotrephes japonensis*); the open stylet groove is slightly visible, which is dorsally covered by the epipharynx (labrum). However, this segment on the ventral side is more developed. In *Cercotmetus* and *Ranatra,* the first segment is incomplete dorsally and invisible; however, there evidently is a stripe on the lateral and ventral side (Figures 8(b) and 9(d)). In the studied species, a condyle (cv) can be observed ventrally, on the distal edge of this segment as documented in: Figures 6(b), 7(f), 8(b), and 9(d).

**Figure 10:**
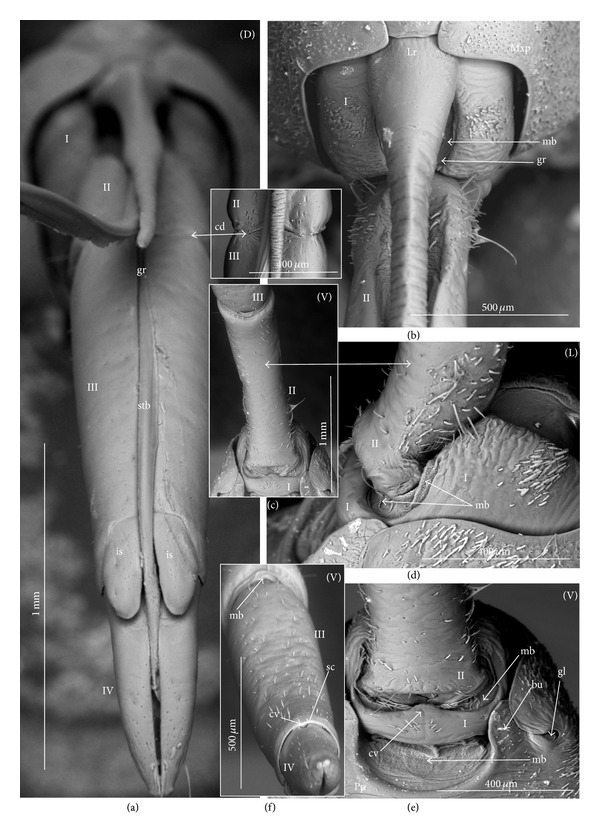
Shapes of the labial segments of the Belostomatidae. (a)–(f) *Belostoma flumineum*. (a) Dorsal view (D) of the labium, all segments are visible, the articulation of the dorsal condyle (cd) between the II and III segments on the dorsal side, (b) the shape of the first segment (I), the stylet groove is opened, the shape of the proximal part of the second segment, the stylet groove is opened, (c) the elongated second segment, ventral view (V), (d) the shape of the first segment (I) in the lateral view (L), the stylet groove is opened, the intersegmental membrane is visible, (e) the midventral condyle (cv) on the distal edge of the first segment, ventral view (V), the intersegmental membrane is distinctly visible, (f) the connection between the third and fourth segments on the ventral side, the midventral condyle (cv) on the third segment is clearly visible. Bu: buccula of the posterior plate, gl: orifice of the maxillary gland, Lr: labrum, Mxp: maxillary plate, Pp: posterior plate of the cranium, stb: stylet bundle.

**Figure 11:**
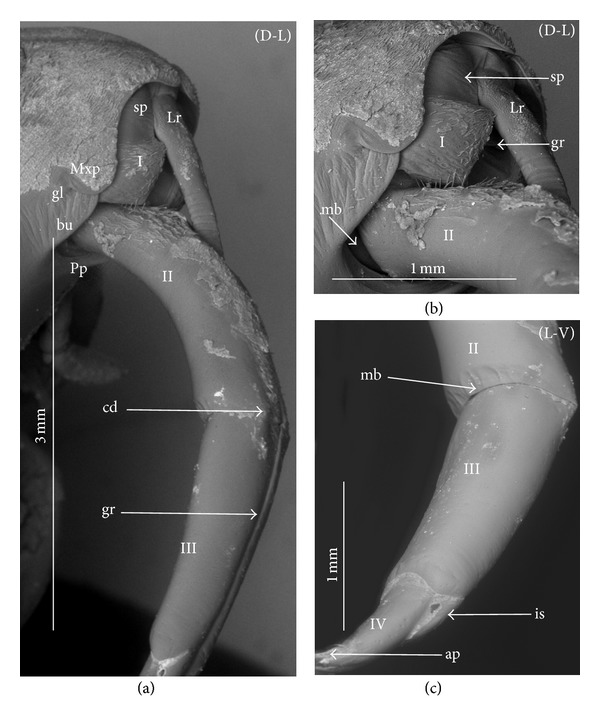
Shapes of the labial segments of the Belostomatidae. (a)–(c).* Deinostoma dilatatum*. (a) Dorso-lateral view (D-L) of the labium, the articulation of the dorsal condyle (cd) between the II and III segments on the dorsal side, the second has the same width proximally and distally, the second segment is slightly longer than the third (b) the shape of the first segment (I) the dorsal side is clearly visible in a slightly rectangular form and the stylet groove is opened, (c) the shapes of the III and IV segments, intercalary sclerites (is) and the apical plate can be observed. Bu: buccula of the posterior plate, gl: orifice of the maxillary gland, Lr: labrum, Mxp: maxillary plate, Pp: posterior plate of the cranium, sp: suspensory plate.

**Figure 12:**
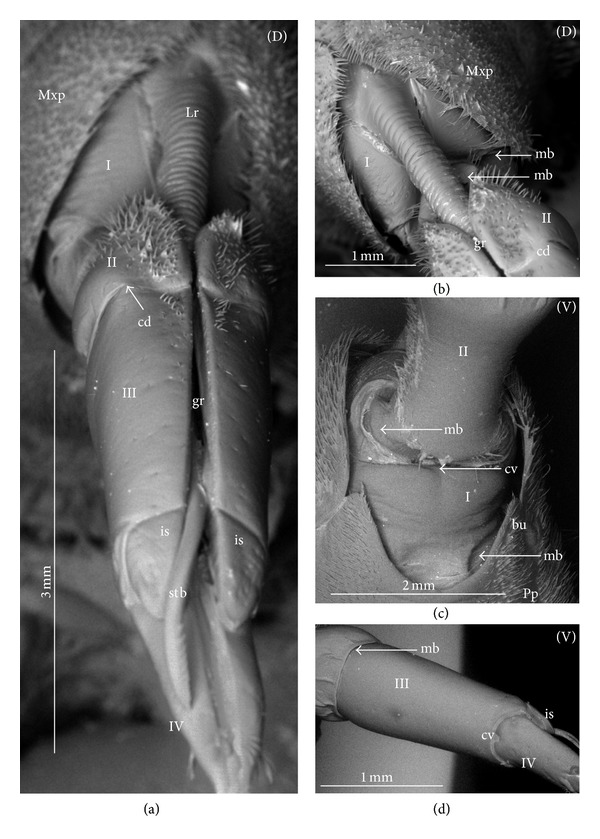
Shapes of the labial segments of the Belostomatidae. (a)–(d) *Hydrocyrius colombiae*. (a) Dorsal view (D) of the labium, all segments are visible, the articulation of the dorsal condyle (cd) between the II and III segments on the dorsal side, the intercalary sclerites (is) and the stylet groove are visible, (b) the shape of the first segment (I) dorsal view of the triangular shape with the distal margin that is curved inside, the stylet groove is opened, the shape of the proximal part of the second stylet groove is opened, the intersegmental membrane is visible, (c) ventral view (V), the first segment with the midventral condyle, the second segment is elongated, (d) the shape of the third segment (III), ventral view (V), the midventral condyle (cv) on the distal edge of the third segment is visible, the intersegmental membranes are distinctly visible. Bu: buccula of posterior plate. Lr: labrum, Mxp: maxillary plate, Pp: posterior plate of the cranium, stb: stylet bundle.

**Figure 13:**
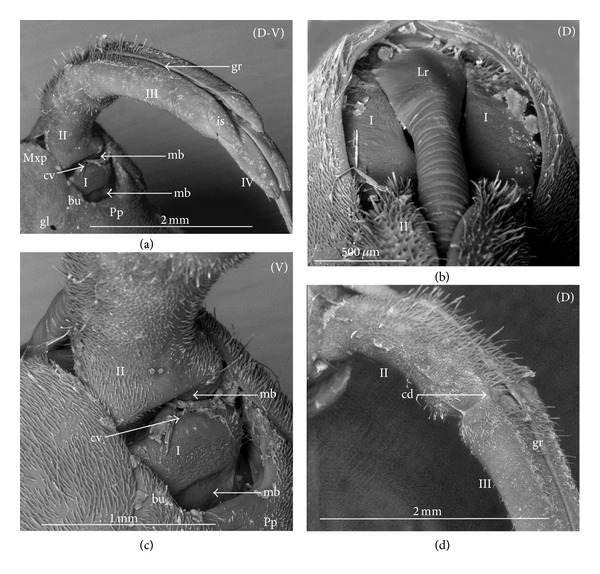
Shapes of the labial segments of the Belostomatidae. (a)–(d) *Limnogeton fieberi*. (a) Dorso-ventral view (D-V) of the labium, all segments and intercalary sclerites are visible (is) (b) the shape of the first segment (I) the dorsal side is clearly visible in a slightly rectangular form and the stylet groove is opened, (c) the shape of the first (I) segment and the proximal part of the second (II) segment, the midventral condyle on the distal edge of the first segment and the intersegmental membrane (mb) are visible, (d) the articulation of the dorsal condyle (cd) between the II and III segments on the dorsal side, the elongated second segment, the second segment has the same width proximally and distally, it is slightly longer than the third. Bu: buccula of posterior plate, gl: orifice of the maxillary gland, Lr: labrum, Mxp: maxillary plate, Pp: posterior plate of the cranium.

**Figure 14:**
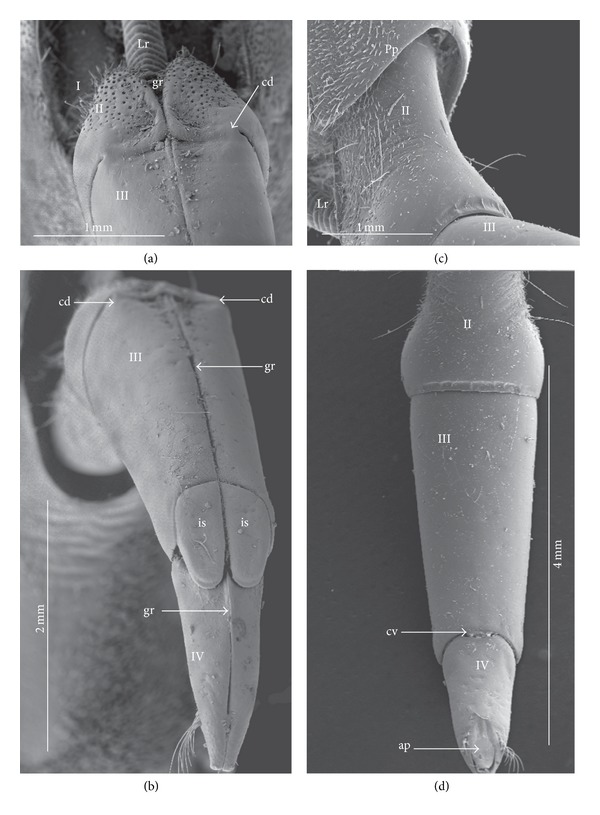
Shapes of the labial segments of the Belostomatidae (Lethocerinae). (a)–(d) *Lethocerus deyrollei*. (a) The shape of the first (I) and the second (II) segments, the stylet groove (gr) is opened, the shape of the proximal part of the second stylet groove is opened, the articulation of the dorsal condyle (cd) between the II and III segments on the dorsal side, (b) dorsal view (D) of the labium, the III and IV segments are visible, the articulation of the dorsal condyle (cd) between the II and III segments on the dorsal side, intercalary sclerites (is) on the III segment, (c) lateral view on the second segment. (d) Ventral view (V), the second segment is short, the third segment is the longest, the connection between the third and fourth segments on the ventral side, the midventral condyle (cv) on the third segment is clearly visible, and the shape of the apical plate. Lr: labrum, Pp: posterior plate of the cranium.

*Belostomatidae.* Belostomatinae: *Belostoma flumineum* (Figures 10(a)–10(f)), *Deinostoma dilatatum* (Figures 11(a)–11(c)), *Appasus major, Hydrocyrius colombiae* (Figures 12(a)–12(d)), *Limnogeton fieberi* (Figures 13(a)–13(d)), Lethocerinae: *Lethocerus deyrollei* (Figures 14(a)–14(d)).

The segment is longer dorsally than ventrally (Figures 10(d) and 10(e)). In the middle, on the dorsal surface, the edges of this segment are not connected (Figures 10(b), 10(d); 11(a), 11(b), and 13(b)) and between them there is a broad stylet groove (gr). Distally, in the middle, the edge of this segment is partly curved inside (ci) (Figures 10(b), 10(d); 11(a), 11(b); and 12(a), 12(b), and 13(b)). Usually, the labrum (Lr) covers the anterior margin of the stylet groove. Externally, this segment is not distinctly visible; it is surrounded by the maxillary plates (Mxp) on the laterodorsal side and ventrally by the posterior plate of the cranium (Pp). Ventrally, on the distal edge of this segment there is formed a condyle (cv) as documented Figures 10(e), 12(c), and 13(c). The ventral surface of this segment is distinctly broader in *Hydrocyrius colombiae* (Figure 12(c)), *Limnogeton fieberi* (Figure 13(c)), and *Appasus major* than in *Belostoma flumineum* (Figure 10(e)). In *Lethocerus* this segment is not clearly visible.

**Figure 15:**
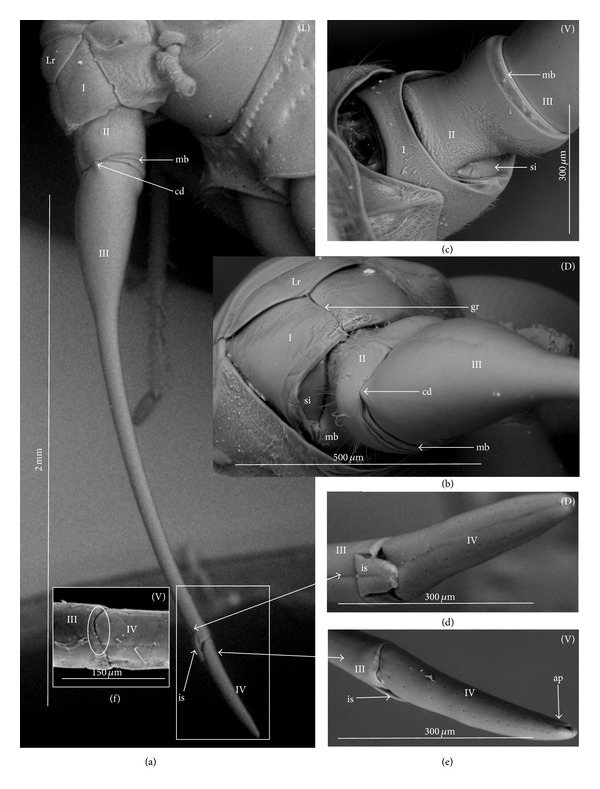
Shapes of the labial segments of the Ochteridae. (a)–(f) *Ochterus piliferus*. (a) Lateral view of the labium, all segments are visible, (b) the square shape of the first segment (I), one intersegmental sclerite (si), the shape of the second segment (II), the dorsal condyle (cd) between the II and III segments on the dorsal side, a wider proximal part of the third segment, ventral view of the intersegmental membrane (mb), (c) the shape of the first (I) and second (II) segments, ventral view (V), lack of the midventral condyle (cv) on the distal edge, one intersegmental sclerite (si), (d) pairs of small intercalary sclerites, the shape of the fourth segment, (e) ventral view, the apical plate (ap) is visible, and (f) the connection between the third and fourth segments on the ventral side, the midventral condyle (cv) is absent (circle). Lr: labrum.

*Ochteridae. Ochterus marginatus*, *Ochterus piliferus* (Figures 15(a)–15(f)). This segment is longer dorsally than ventrally (Figures 14(a) and 14(b)). In the middle, on the dorsal surface of this segment the edges are in contact (Figures 15(a) and 15(b)). From the lateral side to the middle part of the segment, the dorsal sclerite is square-shaped (Figure 15(b)). Underneath there is the stylet groove, invisible on the photograph. The proximal edge of the segment is partly covered by the labrum (Lr). The ventral side is a narrow strip *without the condyle* (Figure 15(c)). The lateral wall is evidently broader than the ventral one.

**Figure 16:**
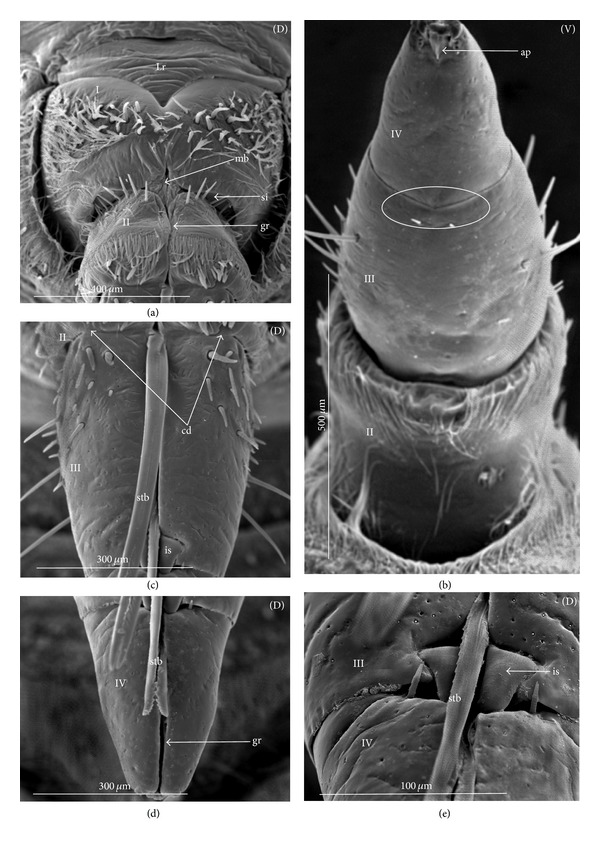
Shapes of the labial segments of the Gelastocoridae. (a)–(e) *Gelastocoris oculatus*. (a) The shape of the first and second segment, dorsal view, the stylet groove is closed, one intersegmental sclerit (si) and intersegmental membrane (mb) are visible, (b) ventral view of the I, II and III segments, the midventral condyle (cv) on the distal edge of III and IV segments is invisible (circle), triangular shape of the apical plate, (c) the shape of the second segment (II), the dorsal condyle (cd) between the II and III segments on the dorsal side, small intercalary sclerites (is), (d) the shape of the IV segment, ventral view (V), the stylet groove is closed, and (e) pairs of the small intercalary sclerites, magnified. Lr: labrum.

**Figure 17:**
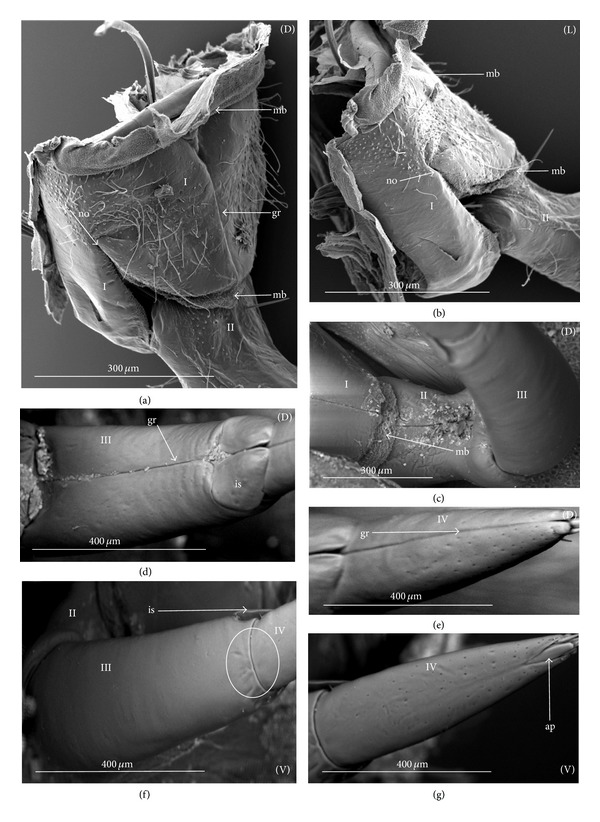
Shapes of the labial segments of the Gelastocoridae. (a)–(g) *Nerthra nepaeformis*. (a) Dorsal view (D) of the labium, the shapes of the I and II segments, a deep notch on the lateral slide, the stylet groove is closed, (b) the shape of the first segment (I), lateral and ventral view, intersegmental membrane (mb) is visible, (c) the shape of the second segment (II)—dorsal view, and the third (III) segment—ventral view, (d) the third segment, dorsal view with the intercalary sclerites, (e) dorsal view of the fourth (IV) segment, (f) no midventral condyle (cv) on the distal edge of the third segment (circle), and (g) ventral view of the fourth segment, the apical plate is visible. Lr: labrum.

**Figure 18:**
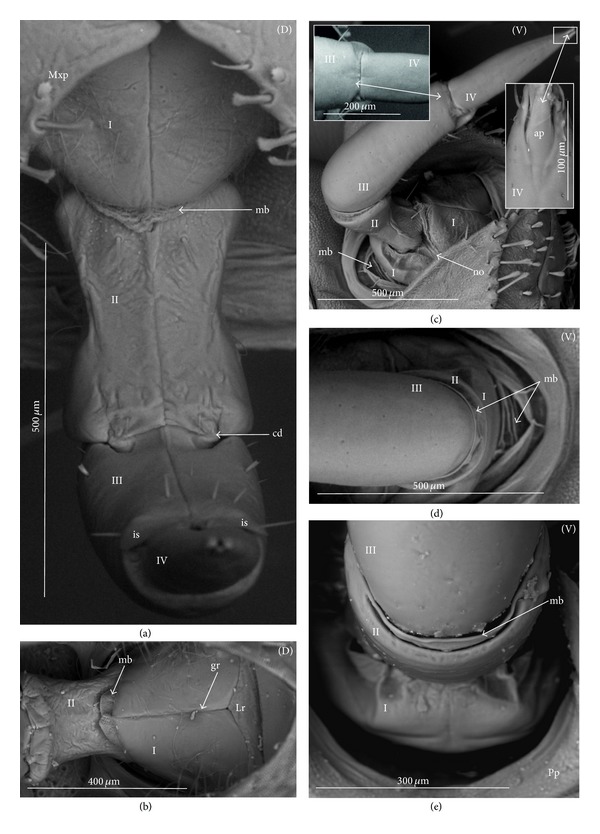
Shapes of the labial segments of the Gelastocoridae. (a)–(e) *Nerthra macrothorax*. (a) Dorsal view (D) of the labium, all segments are visible, the dorsal condyle (cd) between the II and III segments on the dorsal side, intercalary sclerites, (b) slightly triangular shape of the first segment (I), intersegmental membrane (mb), dorsal view, (c) the shape of the first (I) segment with a notch (no), the cuplike second (II) segment, lateral view, no midventral condyle (cv) on the distal edge of the third segment, the shape of the apical plate (ap), (d) the intersegmental membranes connecting the I, II and III segments, the second segment is retracted into the first one, and (e) ventral view, a connection between the first (I) and second (II) segments, no midventral condyle (cv) on the distal edge of the first segment, Lr: labrum, Mxp: maxillary plate, Pp: posterior plate of the cranium.

*Gelastocoridae*. *Gelastocoris oculatus* (Figures 16(a)–16(e)), *Nerthra nepaeformis* (Figures 17(a)–17(g)), and *N. macrothorax* (Figures 18(a)–18(e)). In *Gelastocoris oculatus *(Figure 16(a)), this segment is long dorsally but much shorter ventrally. On the dorsal part of the ring in the middle, the edges are in contact along the entire length. The stylet groove is placed very deep, so it is invisible on the photograph. The base of this segment is covered by a triangular, short labrum. Ventral part of the segment is narrow and slightly hidden when the labium is retracted. In *Nerthra nepaeformis* (Figures 17(a) and 17(b)) and *N. macrothorax* (Figures 18(a) and 18(c)), this segment is modified. Between the dorsal and lateral side, there is a deep (notch) incision (in) reaching half of the length of this segment; the incision is filled with a membrane. Dorsally, the basal part of this segment is wider, but the distal part is slightly narrowed, so this part of the segment is approximately subtriangular (Figures 18(a) and 18(b)). Laterally and ventrally, this segment is slightly undulated and forms an irregular strip. Probably it is slightly sclerotized and the ventral condyle has not formed (Figures 18(d) and 18(e)), so the condyle cannot be observed. In Figures 18(d) and 18(e), the ventral part of this segment is smooth and the second segment is retracted inside of the first one; the lack of the condyle can result in an increased range movement of these segments.

**Figure 19:**
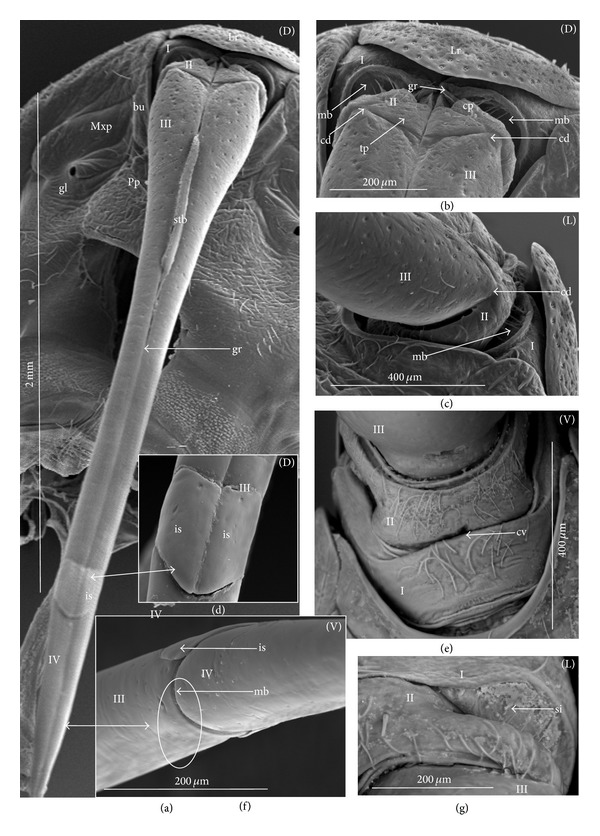
Shapes of the labial segments of the Aphelocheiridae. (a)–(g) *Aphelocheirus aestivalis* (a) Dorsal view (D) of the labium, all segments (I, II, III, IV) are visible, (b) the shape of the first segment (I), the stylet groove (gr) is opened in the proximal part, the dorsal surface of the second segment (II) is divided into the triangular plate (tp) and the convex plate (cp), the second segment (II)—the stylet groove is opened, the articulation of the dorsal condyle (cd) between the II and III segments on the dorsal side, (c) lateral view, the proximal part of the third segment, the intersegmental membrane (mb) is visible, the second segment, ventral (V) view, (d) shapes of the intercalary sclerites (is), (e) the midventral condyle (cv) on the distal edge of the first segment, ventral view (V), the intersegmental membrane is distinctly visible, the shape of the first (I) and second (II) segments, viewed from the ventral side, (f) the connection between third and fourth segments on the ventral side, the midventral condyle (cv) on the third segment is not visible (circle), only the membrane (mb) is present, and (g) an intersegmental sclerit (si) between the first and second segment, lateral view. bu: buccula of the posterior plate, gl: orifice of the maxillary gland, Lr: labrum, Mxp: maxillary plate, Pp: posterior plate of the cranium, stb: stylet bundle.

*Aphelocheiridae* (*Aphelocheirus aestivalis*, Figures 19(a)–19(g)). This segment, is short and almost covered on the dorsal side by a rather short, triangular labrum (Figures 19(a), 19(b) and 19(c)). In the middle of this segment the edges are in contact dorsally and the stylet groove in closed so it is externally invisible. On the ventral side, the segment slightly narrows in comparison to the dorsal side, and the distal edge is equipped with the midventral condyle (Figure 19(e)).

**Figure 20:**
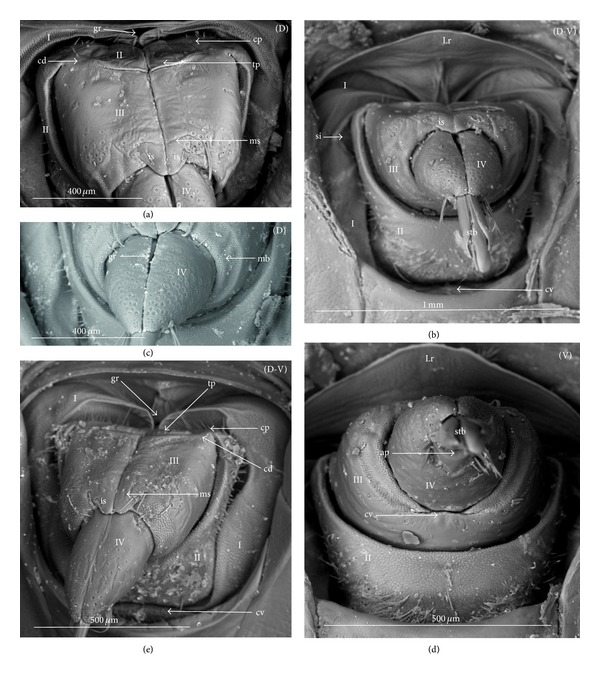
Shapes of the labial segments of the Naucoridae. Cheirochelinae, (a)–(d) *Cheirochela feana,* (e)* Gestroiella limnocoroides*. (a) Dorsal view (D) of the labium, the shape of the first segment (I), the stylet groove (gr) is opened, the dorsal surface of the second segment (II) is divided into the triangular plate (tp) and the convex plate (cp), in the second segment (II) the stylet groove is closed, the articulation of the dorsal condyle (cd) between the II and III segments, the shape of the intercalary sclerites (is), (b) the shape of the segment on the dorsal and ventral side (D-V), the lateral membrane with the intersegmental sclerit (si), the midventral condyle (cv) on the proximal edge of the first segment is visible, (c) the dorsal view, the conical shape of the fourth segment, the intersegmental membrane (mb), the stylet groove is closed (gr), (d) ventral view of the segments, the connection between the third and fourth segments, the midventral condyle (cv) and the apical plate (ap) are visible, and (e) the view from the dorsal and ventral sides, the first segment with the open stylet groove (gr) and with the midventral condyle (cv) on the distal edge, dorsally the surface of the second segment is divided into the triangular plate (tp) and the concave plate (cp), the articulation of the dorsal condyle (cd), the groove is closed in the II, III, and IV segments, the intercalary sclerites are distinct. Lr: labrum, stb: stylet bundle.

**Figure 21:**
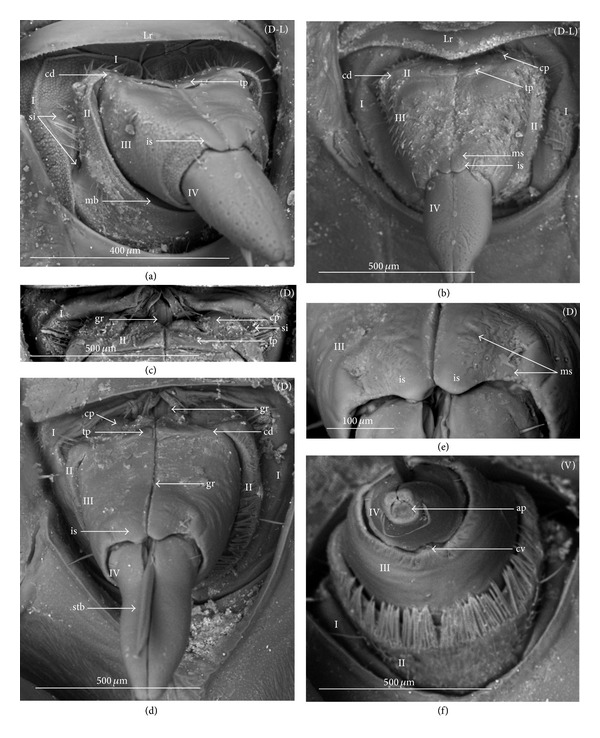
Shapes of the labial segments of the Naucoridae. Cheirochelinae, (a) *Coptocatus oblongulus*, (b)-(c) *Coptocatus kinabalu*, (d)–(f)* Tanycricos longiceps*. (a)-(b) Dorsal and lateral views (D-L) of the labium, the shape of the first segment (I) and intersegmental sclerites (si), the dorsal surface of the second segment (II) is divided into the triangular plate (tp) and the convex plate (cp), in the second segment (II) the stylet groove is closed, the articulation of the dorsal condyle (cd) between the II and III, segments, the shape of the intercalary sclerites (is) with the visible membrane (ms), the conical shape of the IV segment, (c) the dorsal surface of the second segment (II) is divided into the triangular plate (tp) and the convex plate (cp), the lateral membrane with the intersegmental sclerit (si) viewed in detail, (d) the shape of the segment on the dorsal side (D), dorsal view of the first segment with the open stylet groove (gr), the second segment is divided into the triangular plate (tp) and the convex plate (cp), the articulation of the dorsal condyle is distinctly visible, the stylet groove is closed in the II, III and IV segments, the intercalary sclerites are present, the conical shape of the fourth segment, (e) the intercalary sclerites magnified, the membranes of these sclerites are marked, and (f) ventral view on the segments, the connection between the third and fourth segments, the midventral condyle (cv) and the apical plate (ap) can be observed. Lr: labrum, stb: stylet bundle.

**Figure 22:**
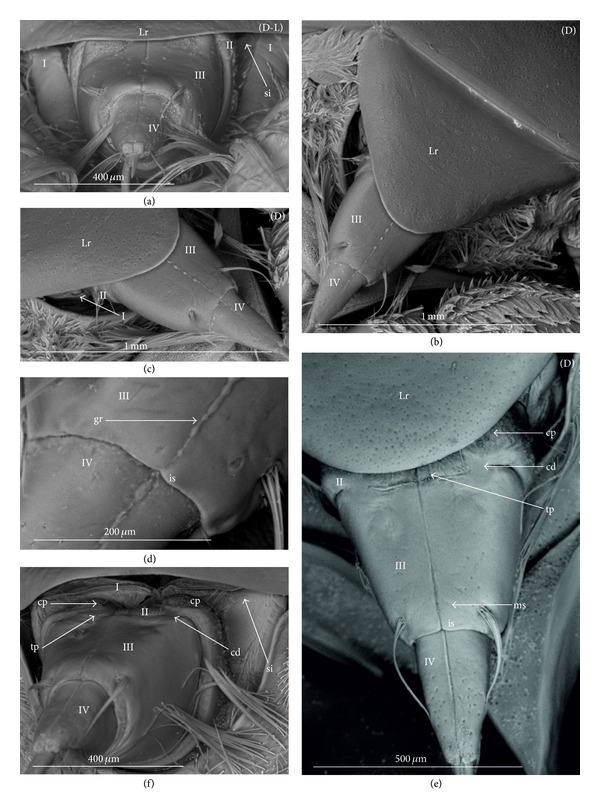
Shapes of the labial segments of the Naucoridae. Laccocorinae, (a)–(d) *Laccocoris hoogstraali*, (e)-(f) *Heleocoris humeralis*. (a)–(c) Dorsal and lateral views (D-L) of the labium, the shape of the first segment (I) and intersegmental sclerites (si), the tubular shape of the third segment (II) and the conical shape of the IV segment, (d) the intercalary sclerites (is) are slightly visible (reduced), (e) dorsal surface of the second segment (II) is divided into the triangular plate (tp) and the convex plate (cp), the intercalary sclerites (is) are slightly visible (reduced), and (f) the shape of the segment on the dorsal side (D), dorsal view of the first segment with the open stylet groove (gr), the second segment is divided into the triangular plate (tp) and the concave plate (cp), the articulation of the dorsal condyle is distinctly visible, the stylet groove is closed in the II, III, and IV segments. Lr: labrum.

**Figure 23:**
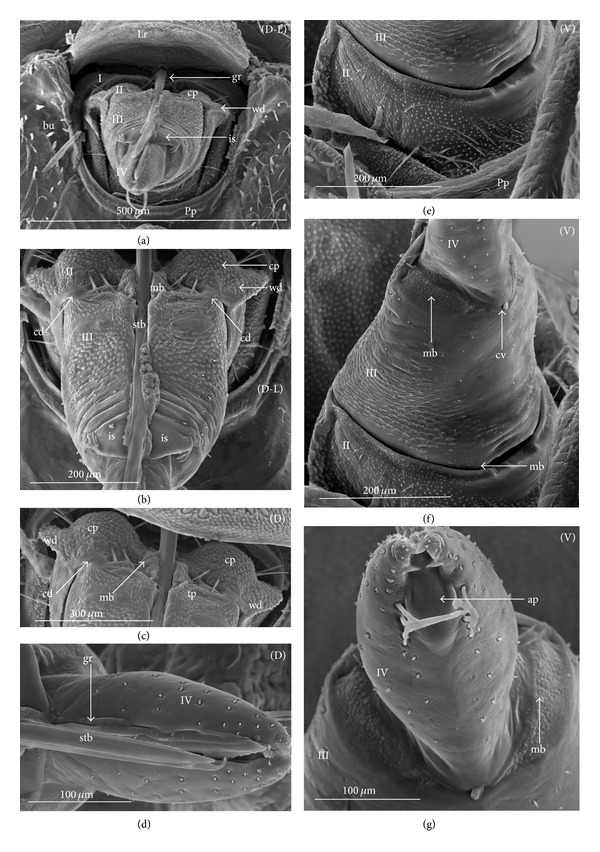
Shapes of the labial segments of the Naucoridae. Limnocorinae, (a)–(g) *Limnocoris lutzi*, (a)-(b) Dorsal and lateral view (D-L) of the labium, the shape of the first segment (I) with the open stylet groove (gr), the second segment dorsally divided into the triangular plate (tp) and the strong convex plate (cp), the wing plate laterally extended (wd), the intercalary sclerites are distinctly visible, the articulation of condyles and the intersegmental membrane are visible, (c) the dorsal surface of the second segment in detailed, (d) the conical shape of the fourth segment, the tubular shape of the third segment (II) and the conical shape of the IV segment, the stylet groove is opened, (e) the connection between the second and third segments on the ventral side (V), (f) the shape of the third segment and its connection with the fourth segment, the midventral condyle is probably situated on the fourth segment, and (g) the shape of the IV segment and the apical plate from the ventral side (V). bu: buccula, Lr: labrum, Pp: posterior plate of the cranium, stb: stylet bundle.

**Figure 24:**
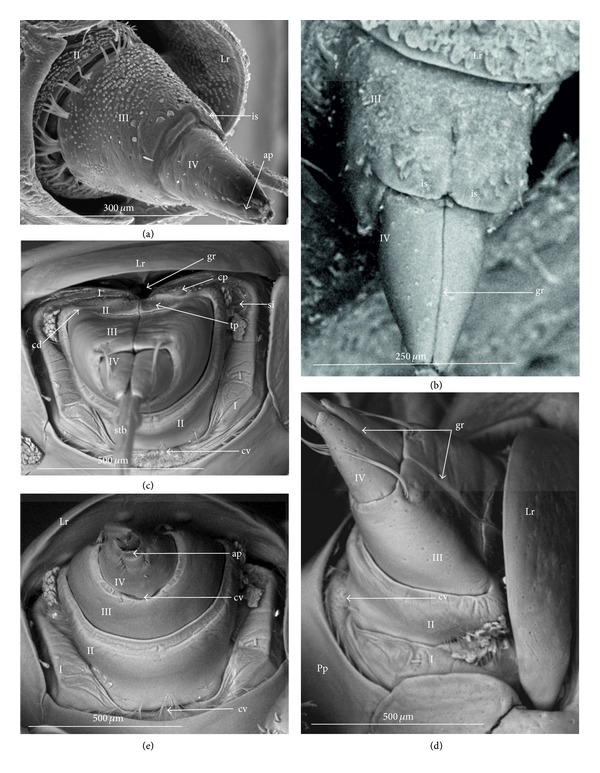
Shapes of the labial segments of the Naucoridae. Cryphocricinae, (a)-(b) *Cryphocricos hungerfordi*, (c)–(e) *Ambrysus occidentalis*. (a) Lateral view (L) of the labium, the first segment (I) is invisible, the part of the second segment is visible, the tubular third and the conical fourth segments are visible, (b) intercalary sclerites (is) are distinctly visible, the stylet groove (gr) is closed, (c) dorsal and lateral view (D-L), in the first segment viewed from the dorsal side the stylet groove (gr) is opened, the second segment is divided into the triangular plate (tp) and the convex plate (cp), the intercalary sclerites are not distinctly visible (reduced), the articulation of condyles and the intersegmental membrane with the intersegmental sclerit (si) are visible, on the ventral side of the first segment the midventral condyle (cv) is present, (d) the shape of the segment in the dorso-lateral view (D-L), the stylet groove is closed in the III and IV segments, the midventral condyle on the first segment is visible, and (e) the shape of the segments, ventral view (V),the midventral condyle situated on the proximal edge of the fourth segment, the apical plate viewed from the ventral side (V). bu: buccula, Lr: labrum, Pp: posterior plate of the cranium.

**Figure 25:**
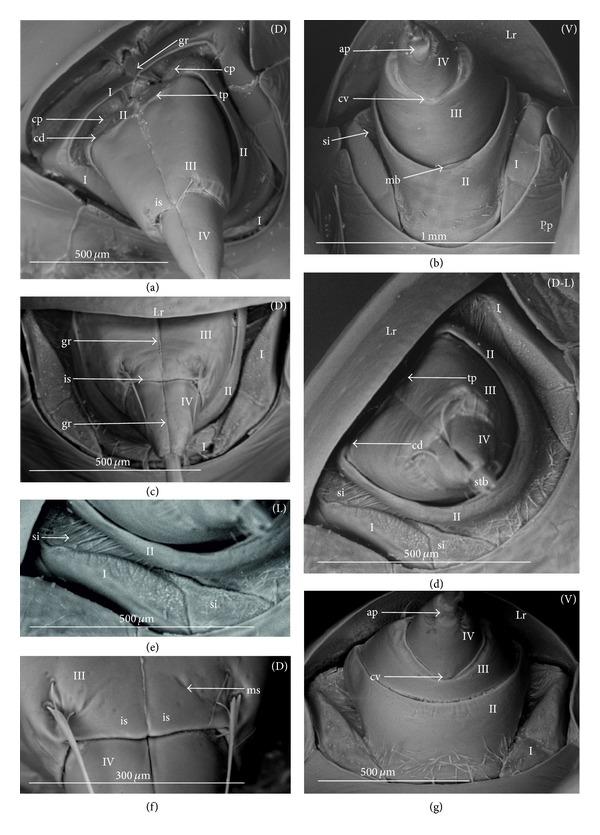
Shapes of the labial segments of the Naucoridae: Naucorinae. (a)-(b) *Ilyocoris cimicoides*, (c)–(g) *Pelocoris femoratus*. (a) Dorsal view (V) of the labium, the first segment (I) with the open stylet groove (gr), the second segment is divided into the triangular plate (tr) and the convex plate (cp), intercalary sclerites (is) are distinctly visible, the stylet groove (gr) is closed in the II, III and IV segments, the articulation between the second and third segments (cd), (b) ventral view of the shape of the segments (V), the midventral condyle is situated on the proximal edge of the fourth segment, the apical plate viewed from the ventral side (V), the intersegmental sclerit (si) can be observed, (c) the shape of the segments in the dorsal view (D), intercalary sclerites are visible, the conical shape of the IV segment, the elements of the first segment (lateral and ventral sides), (d) dorsal and lateral views (D-L), the second segment is divided into the triangular plate (tp) and the convex plate, the articulation of condyles and the intersegmental membrane with two intersegmental sclerites (si) are visible, on the ventral side of the first segment the midventral condyle (cv) is present, (e) intersegmental sclerites (si) magnified, (f) the intercalary sclerites are distinctly visible, the membrane (ms) is slightly visible, and (g) the shape of the segments, ventral view (V), the midventral condyle is situated on the proximal edge of the fourth segment, ventral view of the apical plate (V). Lr: labrum, Pp: posterior plate of the cranium.

**Figure 26:**
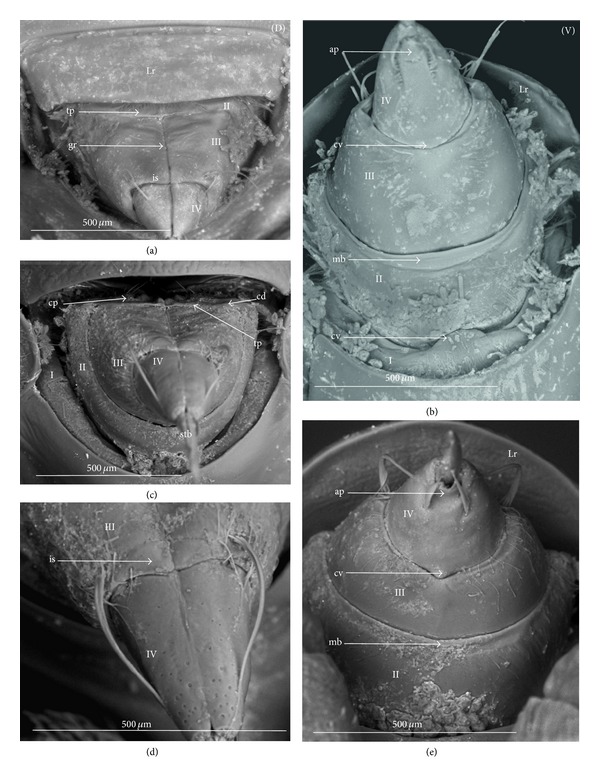
Shapes of the labial segments of the Naucoridae. Naucorinae, (a)-(b) *Macrocoris rhantoides*, (c)–(e) *Neomacrocoris handlirschi*. (a) Dorsal view (D) of the labium, the first segment (I) invisible, the second segment with the triangular plate partly visible, the stylet groove in the II, III and IV segments is closed, (b) ventral view (V), the first segment with the midventral condyle (cv) and the proximal edge of the fourth segment, the apical plate is present, (c) the shape of the segments, dorsal view (D), the second segment is divided into the triangular plate (tr) and the convex plate (cp), the articulation between the second and third segments (cd), (d) the intercalary sclerites (is) are slightly visible, and (e) ventral view (V), the proximal edge of the fourth segment possess the midventral condyle (cv), the apical plate can be observed. Lr: labrum, stb: stylet bundle.

**Figure 27:**
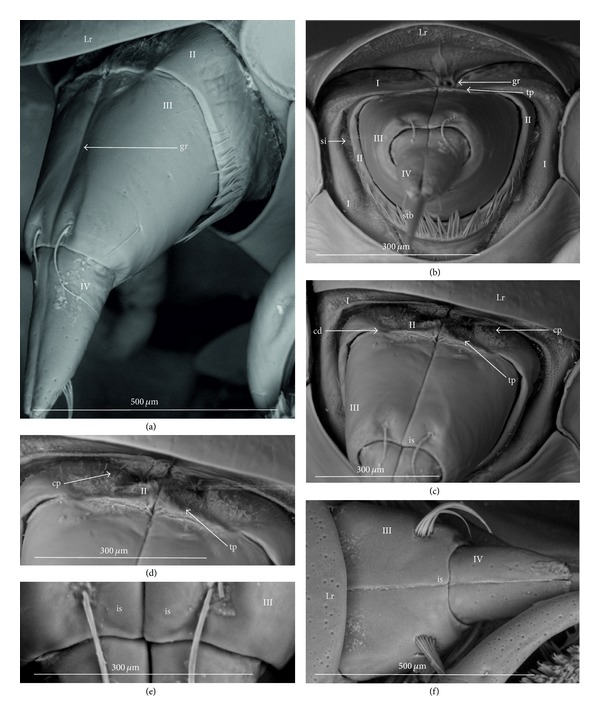
Shapes of the labial segments of the Naucoridae. Naucorinae, (a)–(e) *Naucoris maculatus*, (f)* Namtokocoris siamensis*. (a) Lateral view on the labium, the II segment has the shape of a narrow strap, the III segment is tubular, the IV segment is conical, the stylet groove is closed in these segments, (b) dorsal view (D) of the labium, the first segment (I) with the open stylet groove (gr), in the second segment the triangular plate is visible, laterally, the intersegmental sclerit (si) can be observed, (c) the shape of the segments in the dorsal view (D), the second segment is divided into the triangular plate (tr) and the convex plate (cp), intercalary sclerites are slightly visible, the articulation between the second and third segments (cd), (d) dorsal view (D), the second segment is divided into the triangular plate (tp) and the concave plate, detailed view, (e) the intercalary sclerites are slightly visible, and (f) dorsal view of the labium, the III and IV segment are visible, the intercalary sclerites (is) are almost reduced. Lr: labrum, stb: stylet bundle.

*Naucoridae*. Cheirochelinae: *Cheirochela feana* (Figures 20(a)–20(d)), *Gestroiella limnocoroides* (Figure 20(e)), *Coptocatus oblongulus* (Figure 21(a)), *Coptocatus kinabalu* (Figure 21(c)), *Tanycricos longiceps* (Figures 21(d)–21(f)); Laccocorinae: *Laccocoris hoogstraali* (Figures 22(a)–22(d)), *Heleocoris humeralis* (Figures 22(e)-22(f)); Limnocorinae: *Limnocoris lutzi* (Figures 23(a)–23(g)); Cryphocricinae: *Cryphocricos hungerfordi* (Figures 24(a) and 24(b)), *Ambrysus occidentalis* (Figures 24(c)–24(e)); Naucorinae: *Ilyocoris cimicoides* (Figures 25(a) and 25(b)), *Pelocoris femoratus* (Figures 25(c)–25(g)), *Naucoris maculatus* (Figures 27(a)–27(e)), *Namtokocoris siamensis* (Figure 27(f)). *Macrocoris rhantoides* (Figures 26(a) and 26(b)), *Neomacrocoris handlirschi* (Figures 26(c)–26(e)). In the proximal and middle part of the segment, on the dorsal side, the edges are not in contact and between the edges (right and left) the stylet groove is open. In the distal part of this segment the edges are in contact and the stylet groove is closed. On the ventral side, the segment narrows in comparison to the dorsal side and possesses a midventral condyle on the distal edge. The first segment has the same shape in all species investigated within the five subfamilies.

**Figure 28:**
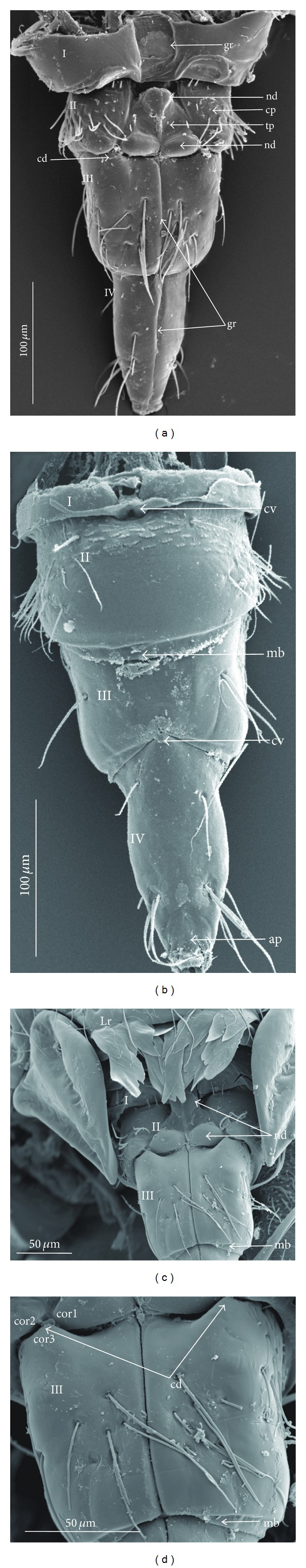
Shapes of the labial segments of the Pleidae. (a)–(d) *Paraplea frontalis*. (a) Dorsal view of the labium, the first segment is a narrow strap with the open stylet groove (gr), the second segment is divided into the triangular plate (tp) and the convex plate (cp), the triangular plate possess two pairs of nodules (no), the III segment is tubular, the IV segment is conical, the stylet groove is closed in these segments, (b) ventral view (V) of the labium, on the distal edge of the first segment (I) and the proximal edge of the fourth segment there is the midventral condyle (cv), the triangular apical plate can be observed, (c) the shape of the II segment, detailed view (D), the proximal part (no) of the triangular plate is slightly raised, and (d) the articulation between the second and third segments (cd) consists of three corners (condyles), the cor3 is distinctly protruding, there are no intercalary sclerites on the third segment (loss).

*Pleidae*. *Paraplea frontalis* (Figures 28(a)–28(d)). This segment is shorter than the other three. The edges on the dorsal side are not in contact along their whole length and the stylet groove is wide and open (Figure 28(a)). The groove is covered only by the labrum. On the ventral side, the segment is two times narrower than on the dorsal side and equipped with the midventral condyle (Figure 28(b)).

**Figure 29:**
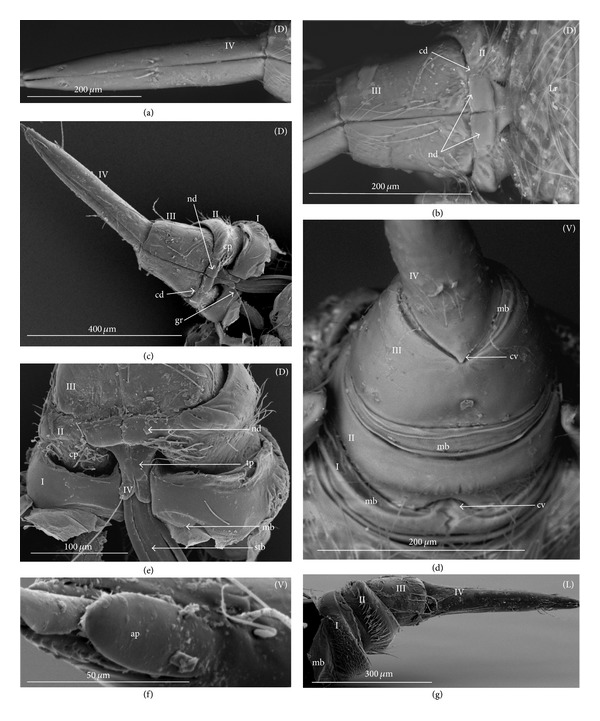
Shapes of the labial segments of the Helotrephidae. (a)–(g) *Hydrotrephes balnearius*. (a) Dorsal view of the labium, the shape and size of the IV segment (this segment is longer than the remaining three), (b) the shape of the second segment (a triangular plate with evidently raised nodules in the distal part of the triangular plate) and the tubular third segment, (c) dorsal view of the labium, the first segment (I) is a narrow strap with the open stylet groove (gr), the second segment is divided into the triangular plate (tp) and the concave plate (cp), the triangular plate features pairs of nodules (no), the tubular III segment, the articulation between the second and third segments (cd), the IV segment has a conical shape, the stylet groove is closed in these segments, (d) ventral view (V) of the labium, on the distal edge of the first segment (I) and the proximal edge of the fourth segment there is the midventral condyle (cv), (e) the I and II segments magnified, (f) the oval apical plate can be observed, and (g) lateral view of all segments (the fourth segment is the longest). Lr: labrum, stb: stylet bundle.

*Helotrephidae*. *Helotrephes semiglobosus, Hydrotrephes visayasensis, Hydrotrephes balnearius *(Figures 29(a)–29(g)) and *Tiphotrephes indicus*. The segment is almost as narrow dorsally as it is ventrally (Figure 29(g)). The edges on the dorsal side are not in contact along their whole length and the stylet groove (with the stylet bundle) is wide and open. The groove is covered by the labrum (Figure 29(b)) and partly by the triangular plate of the second segment (Figures 29(c) and 29(e)). The midventral condyle is present (Figure 29(d)).

**Figure 30:**
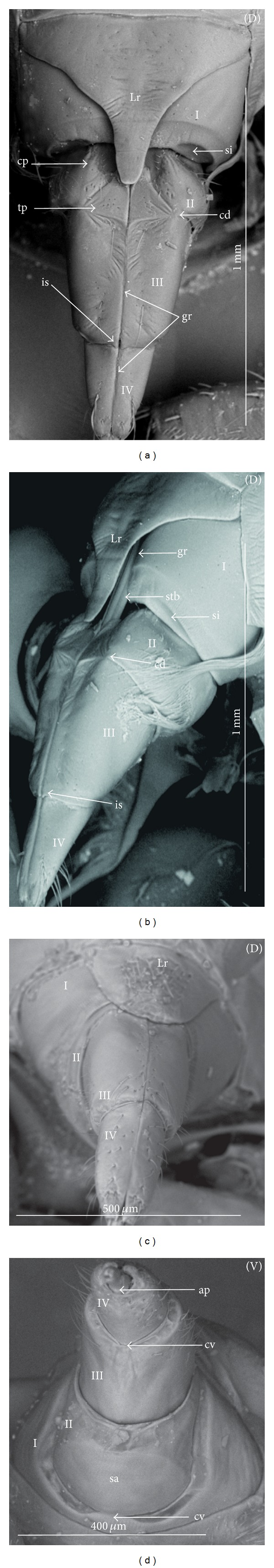
Shapes of the labial segments of the Notonectidae. Anisopinae. (a)-(b) *Anisops camaroonensis*, (c)-(d) *Buenoa uhleri*. (a) Dorsal view of the labium, the shape of the second segment—the planar triangular plate (tp) and the slightly convex plate (cp), the third segment is tubular, the fourth segment is conical, the articulation between the second and third segments (cd), the intersegmental sclerit (si) is visible on the membrane of the first segment, the stylet groove (gr) in the III and IV segments is closed, (b) lateral view of the segments, the first segment and the base of the second segment each have an open a stylet groove (gr), (c) dorsal view of the labium, the first segment (I), the second (II) segment is invisible (covered by the labrum), the III segment is tubular, the IV segment is conical, the stylet groove is closed in these segments, and (d) ventral view (V) of the labium, on the distal edge of the first segment (I) and proximal edge of the fourth (IV) segment there are midventral condyles (cv), the apical plate can be observed, the second segment on the ventral side is evidently modified—the convex sack (sa) can be observed. Lr: labrum, stb: stylet bundle.

**Figure 31:**
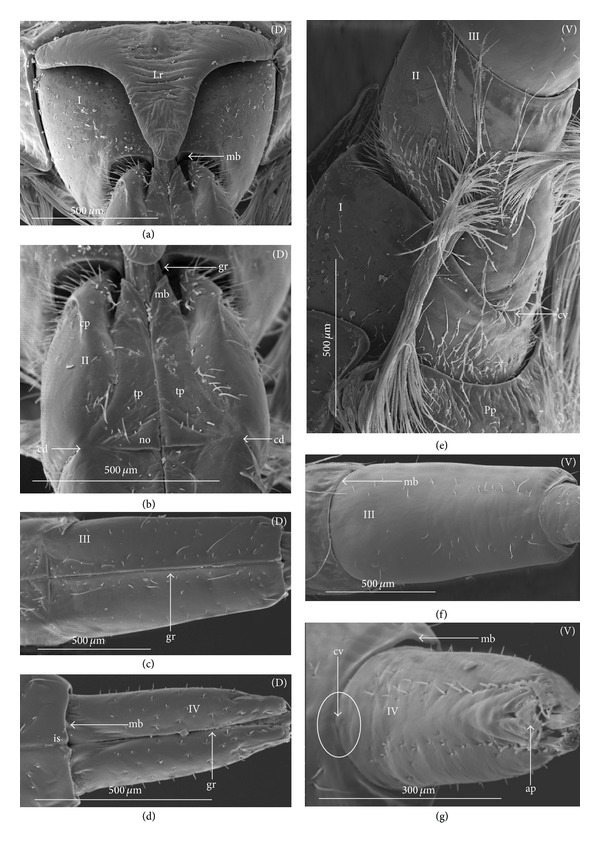
Shapes of the labial segments of the Notonectidae. Notonectinae. (a)–(d). *Notonecta glauca*. (a) Dorsal view on the segments: the first segment is wide, the stylet groove is covered by the labrum, the base of the second segment possesses the open stylet groove (gr), (b) dorsal view of the labium, the shape of the second segment is that of a triangular plate (tp) with a distinctly visible nodule (no) and a convex plate (cp), the articulation between the second and third segments (cd), (c) the third segment is tubular, the stylet groove (gr) is closed, (d) the fourth segment is conical, the stylet groove (gr) is closed, (e) ventral view (V) of the segments, the first segment on the distal edge has a midventral condyle (cv), this segment is narrower ventrally than dorsally, (f) ventral view (V), the III segment is tubular, and (g) the IV segment is conical, the stylet groove is closed in this segment, on the fourth segment there is situated the midventral condyle (cv) but it is only slightly visible (circle), the apical plate can be observed. Lr: labrum, Pp: posterior plate of the cranium.

**Figure 32:**
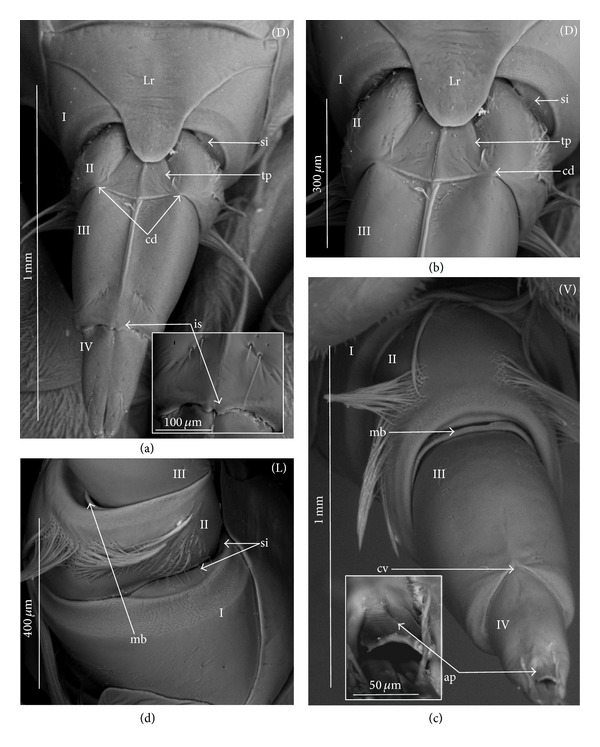
Shapes of the labial segments of the Notonectidae. Notonectinae. (a)–(d)* Enithares bergrothi*. (a) Dorsal view of the shape of the segments (I, II, III and IV), the first segment is wide, the stylet groove is covered by the labrum, the shape and size of the reduced intercalary sclerites, the stylet groove (gr) in the III and IV segments is closed, (b) dorsal view (V) of the segments in detail, the second segment is divided dorsally into the triangular plate (tp) and the convex plate (cp), the articulation between the II and III segments is visible, (c) ventral view (V), the shape of the segments, the midventral condyle on the proximal edge of the fourth segment is visible, the trapezoidal apical plate can be observed, the membranes (mb) are visible, and (d) lateral view, the first segment is narrower ventrally than dorsally, the second segment is narrower dorsally than ventrally, the intersegmental sclerites can be observed. Lr: labrum.

**Figure 33:**
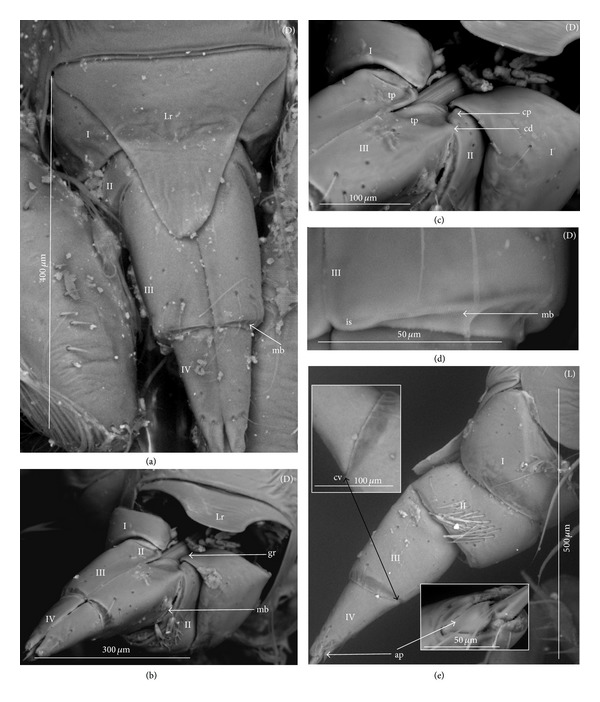
Shapes of the labial segments of the Notonectidae. Notonectinae. (a)–(e) *Nychia sappho*. (a) Dorsal view of the shape of the segments (I, II, III, and IV), the first segment is wide, the stylet groove is covered by the labrum, (b) dorsal view of the labium, the first segment with the open t stylet groove (gr), the second segment, at the base of this segment the stylet groove is opened, the third segment is tubular, he fourth segment is conical, the stylet groove (gr) in III and IV segments is closed, (c) dorsal view (V) of the segments in detail, the second segment is divided dorsally into the triangular plate (tp) and the convex plate (cp), the articulation between the II and III segments is visible, (d) the shapes and sizes of the reduced intercalary sclerites, and (e) lateral view, the first segment has a midventral condyle (cv) on the distal edge, this segment is narrower ventrally than dorsally, the second segment is narrower dorsally than ventrally, the III segment is tubular, the IV segment is conical, on the fourth segment there is the midventral condyle (cv) but it is only slightly visible, the trapezoidal apical plate (ap) can be observed. Lr: labrum.

*Notonectidae*. Anisopinae: *Anisops camaroonensis* (Figures 30(a) and 30(b)), *Anisops sardea*, *Buenoa uhleri* (Figures 30(c) and 30(d)) Notonectinae: *Notonecta glauca* (Figure 31(a)–31(g))*, Enithares bergrothi *(Figures 32(a)–32(d)), *Nychia sappho *(Figures 33(a)–33(e)). The segment is well developed; it is slightly wider dorsally than ventrally (Figures 30(b), 31(a), 31(b), 32(a), 32(d), 33(b), 33(c), and 33(b)). The stylet groove is wide and edges are not in contact in the middle of the dorsal part (Figures 30(b), 31(b), 33(c), and 33(d)). The groove is covered by the labrum. The labrum is triangular and narrowed and reaches up to the second segment in *Anisops* (Figures 30(a) and 30(b)), *Notonecta *(Figure 31(a)) and *Enithares *(Figures 32(a) and 32(b)). However, it is longer in *Buenoa* (Figure 30(c)) and *Nychia* (Figure 33(a)), reaching up to the third segment. The midventral condyle (cv) is present (Figures 30(d) and 31(e)).

### 3.5. External Variation of the Second Segment

The dorsal surface of the second segment represents two structural variations among the nine families studied; thus, this character is presently analyzed with respect to groups of families.

*Second Segment*. The dorsal surface is not divided into the Nepidae, Belostomatidae, Ochteridae, and Gelastocoridae. The dorsal side is a uniform plate situated symmetrically on both sides (right and left) of the centrally placed stylet groove. In the proximal part, the edges of this segment are not in contact in the middle and there is no definite stylet groove in the Nepidae and Belostomatidae. It is placed near the apex of this segment as the closed stylet groove.

In general appearance, this segment has approximately a cuplike-shape (the basal part is narrow and the distal part is wider), which is distinctly visible on ventral side of this segment in the Nepidae, in some of the Belostomatidae (*Lethocerus*, *Hydrocyrius*) as well in the Ochteridae. Furthermore, this segment can have a more cylindrical shape (the width of the basal and proximal part is almost the same) as can be observed in the Belostomatidae (*Belostoma*,* Deinostoma*, and *Limnogeton*) and Gelastocoridae.

In the distal part, on the dorsolateral side there are formed points of articulation. In the Nepidae (Figures 7(a); 8(a); 9(b); and 9(c)) and Belostomatidae (Figures 10(a); 11(a); 12(a); 13(d); 14(a) and 14(b)), particular elements of this articulation (cd) are not clearly visible, and a band is formed between the third and second segments at the dorsolateral side. 

Probably, another type of articulation is present in the Ochteridae (Figure 15(b)). The mechanism consists of one condyle (cd) of the third segment and a slightly concave one on the surface of the second segment. In addition, in the Nerthrinae (Figure 18(a)), the band of the articulation is yet more complex.

*Second Segment. The dorsal surface is divided into two areas.* On the dorsal side, in the middle of this segment there are two triangular plates (tp), each placed on both sides of the closed stylet groove. The proximal part of the triangular plate is narrower than the distal one. Laterally to them, there is attached the remaining part of the segment. This part is more convex (cp) than the triangular plate. The triangular plate is divided from the rest by a membrane. The wider part of the triangular plate is in contact with the proximal edge of the third segment. The dorsal surface divided in such a way has been found in the Aphelocheiridae (Figures 19(a), 19(b), and 19(c)), Naucoridae (Figures 20(a), 20(e), 21(a), 21(b), 21(c), 21(d), 22(f), 23(a), 23(b), 23(c), 24(c), 24(d), 25(a), 25(d), 26(a), 26(c), 27(b) and 27(c)), Pleidae (Figures 28(a), and 28(c)), Helotrephidae (Figures 29(b), 29(c), and 29(e)), and Notonectidae (Figures 30(a), 30(b), 31(b), 32(a), 32(b), and 33(c)). However, several distinct characters have been observed in particular taxa. In the Limnocorinae (*Limnocoris lutzi*, Figures 23(a), 23(c), and 23(e)), the second segment on the laterodorsal side has a large dome-shaped wing (wd) and the proximal part is convex (cp), evidently strongly raised contrary to the remaining species. In addition, in the Pleidae (Figure 28(a)) and Helotrephidae (Figure 29(e)), there has been observed an even more divided triangular plate (tp). Distal part of this area is clearly separated from the remaining part, forming a node (nd, nodes). However, in the Notonectidae (Figure 31(b)), these nodes are not as large as in the Pleidae and Helotrephidae.

In the Pleidae and Helotrephidae, on the dorsolateral surface between the second and third segments, there are visible three areas, that is, a corner (cor1) of the triangular plate, a corner (cor2) of the convex part of the segment, and a corner (cor3) of the dorso-lateral part of the third segment. Together, they form the point (cd) of articulation specific to these families (Figure 28(d)). This point of articulation (cd) is placed symmetrically, so on the dorsal side there are two points of articulation (left and right). In the Naucoridae and Notonectidae the type of articulation is similar to that encountered in most other families (except for the characteristic articulation in the Ochteridae and Nerthra).

### 3.6. Tubular and Conical Shapes of the Third and Fourth Segments

The third segment is tubular (the base and the apex have the same width) and on the dorsal side it has intercalary sclerites, while the fourth segment has a conical shape; that is, the basal part is wider than the apex.

In the Nepidae and some of the Belostomatidae (*Hydrocyrius*, *Limnogeton,* and *Lethocerus*) and Gelastocoridae the third labial segment is relatively the longest of the four. Only in the *Belostoma* and *Deinostoma,* the second segment is slightly longer than the third. The significant length of the third segment is characteristic for the Ochteridae, Nerthrinae, and Aphelocheiridae. In the Naucoridae, Pleidae, and Notonectidae these segments are of similar length as the fourth segment except for the Helotrephidae, in which the fourth segment is the longest. 

A characteristic element of the third segment is the fact that the midventral condyle (cv), positioned on the distal edge of this segment, fits into the concave on the proximal edge of the fourth segment, thus connecting the two segments. Such a condyle has been observed in the Nepidae (*Borborophes mayri* Figure 6(b), *Laccotrephes japonensis* Figure 7(c), *Cercotmetus asiaticus* Figure 8(b), and *Ranatra chinensis* Figure 9(d)), Belostomatidae (*Belostoma flumineum* Figure 10(f), *Hydrocyrius colombiae* Figure 12(d), *Lethocerus deyrollei* Figure 14(d)) and Naucoridae (Cheirochelinae, *Gestroiella limnocoroides* Figure 20(d), and *Tanycricos longiceps* Figure 21(f)). 

Another, the second type of the connection between the third and fourth segment is realized probably only through a membrane, because no midventral condyle has been observed in these segments in the Ochteridae (Figure 15(e)), Gelastocoridae (*Gelastocoris oculatus* Figure 16(b), *Nerthra nepaeformis* Figure 17(g), *N. macrothorax* Figure 18(c)), and Aphelocheiridae (Figure 19(f); circle: this condyle was not observed).

Moreover, there is a third type of the connection, represented by the midventral condyle situated on the proximal edge of the fourth segment in some Naucoridae (Limnocorinae *Limnocoris lutzi* Figure 23(f), Cryphocricinae *Ambrysus occidentalis* Figure 24(e); Naucorinae: *Ilyocoris cimicoides* Figure 25(b), *Pelocoris femoratus* Figure 25(g), *Macrocoris rhantoides*, Figure 26(b); *Neomacrocoris handlirschi*, Figure 26(e)) Pleidae (Figure 28(b)), Helotrephidae (Figure 29(d)) and Notonectidae (*Buenoa uhleri* Figure 30(d), *Notonecta glauca* Figure 31(g), *Enithares bergrothi* Figure 32(c)).

## 4. Discussion

### 4.1. Structural Diversity of the Labium of the Nepomorpha

The Nepomorpha (Hydrocorisae, Cryptocerata) represent some examples of modified labium in particular taxa that are evidently separated from the Corixidae. Parsons [20] has shown that in the coroixids the structures of the labium have been subject to unusual changes and homologies of the labial structures between them and the other nepomorphans are difficult to identify.

The present study describes several new, external, morphological characters of the labium in representatives of the nepomorphan taxa. Previous studies have demonstrated the morphological heterogeneity of the internal and external labial structures across different nepomorphan groups [9, 12, 18, 20, 21, 25]. There have been identified several inconsistencies as well as several congruences between previous descriptions of the external characters of the labium of the Nepomorpha and those presented here. However, due to a detailed and extensive examination of a number of different species, the present study has enriched the available data on this subject.

An interesting morphological diversity begins to emerge from the present study and several features appear to be of interest for further investigation. The obtained data should be taken into account in future cladistic analysis. Additionally, the necessity of their use in further phylogenetic analysis is supported by the fact that in the last combined phylogenetic analysis (using morphological and molecular data) conducted by Hebsgaard et al. [30] only four characters of the labium were incorporated.

Presently, the interpretation of the external morphology of the labial segments of nepomorphan taxa is based on a dozen or so species showing 27 informative characters (apomorphic) which have been evaluated from the evolutionary perspective in the different subfamilies or families. These characters of the labial segments have been evaluated in relation to the characters of gerromorphans described by Andersen [33]. The Gerromorpha have been established as an outgroup for the Nepomorpha [34], and they have also been used for polarization of the nepomorphan characters by Hebsgaard et al. [30].

### 4.2. A Comparison of the Apical Plates across the Nepomorpha

The apical plate of nepomorphans has been found in five differing forms (oval, palm-shaped, triangular, trapezoid, and rectangular) unlike the one type of the labial plate or apical plate described by Quadri [21], Parsons [20], Popov [9], and Cobben [18]. The newly detected shapes of the apical plate are characteristic of different (sub-)families. The palm-shaped apical plate is present only in the Belostomatinae and Lethocerinae; a similar shape has also been found in the Nerthrinae; however in that group it is evidently narrower (the shape of a slim palm). The oval shape of the apical plate of various sizes occurs in the nepids, ochterids, aphelocheirids, limnocorines, and helotrephids. A similar shape, however, distinctly smaller, can be observed in the Corixoidea (Brożek, submitted, 2013). In fact the apical plate in the plesiomorphic taxa, such as the Belostomatidae and Nepidae [9, 20], occurs in two shapes and it is problematic which one is plesiomorphic and which is apomorphic. It might be assumed that the oval plate in the Nepidae has a plesiomorphic condition, which is suggested by the fact that it is almost identical with the form of the apical plate described as a finger-nail shape, which has been evidenced in the Gerromorpha (an outgroup for the Nepomorpha, presented by Wheeler et al. [34]), as well as in other groups of heteropterans by Cobben [18]. The presence of plesiomorphy evidenced in those groups can also be found in several other nepomorphan families studied here.

Despite these uncertainties, a synapomorphy can be pinpointed (i.e., the palm-shaped apical plate) for the Belostomatinae and Lethocerinae and possibly also an autapomorphy for the Belostomatidae; however, the representatives of the Horvathiniinae (the third subfamily) have not been reviewed. An obvious autapomorphy (slim-palm shape of the apical plate) has been estimated for the Nerthrinae. The latter group systematically belongs to the Gelastocoridae [9, 32, 35, 36] and is regarded as the most diverse of the two subfamilies, showing several various autapomorphies. Presently yet another autapomorphy has been found for that group. Apart from the Nerthrinae, another shape of the apical plate (triangular plate) has been exhibited in the second subfamily Gelastocorinae and in addition in a systematically distant family Pleidae. In such a case, such a character is treated as a convergence. The recognition of the rectangular shape of the apical plate in the Naucoridae (except for the Limnocorinae) and the recognition of the trapezoidal shape of the apical plate in the Notonectidae (Anisopinae and Notonectinae) have been estimated as separate autapomorphies in these taxa. 

In the previously discussed nepomorphan taxa based on this set of characters there cannot be found a total compliance of the relationships of (sub-)families on the pattern of close relationships indicated in the recent hypotheses [12, 30, 31]. However, this set of characters brings four apomorphies (Table 3) and informs only that the Belostomatinae and Lethocerinae are a sister group, similar to the case of the relationship of the Anisopinae and Notonectinae; it supports the monophyly of the Nerthrinae as well as of the Naucoridae except for that of the Limnocorinae. In the latter group, this character can rather be regarded as the reverse state. 

In most heteroptera taxa, the apical plate is relatively preserved in different shapes (in the Gerromorpha, unpublished data, Brożek); however, a careful comparative study has not been conducted in this area so far. Cobben [18] has reported that apical plates can be found in several taxa such as the Saldidae, Microphysidae, Dipsocoridae, Enicocephalidae, Reduviidae, Nabidae, Miridae, and all Gerromorpha. However, in many species of the Pentatomomorpha (*Graphosoma*, *Pentatoma*, *Dolycoris*, *Acanthocoris*, and *Neides*) and in *Piesma* the inspection has definitely revealed no presence of the apical plate. However, a more detailed inspection carried out in the future may yet reveal the presence of small (vestigial) apical plates in those species.

### 4.3. A Comparison of the Intercalary Sclerites across the Nepomorpha

The intercalary sclerites occur in various shapes in most groups of the Nepomorpha, although in several groups they are greatly reduced (Laccocorinae, Naucorinae, and Notonectidae) or completely lost, as in the Pleidae and Helotrephidae.

The modification appears to be related both to the general shapes and sizes (large, medium, and small) of intercalary sclerites of these bugs. The large and medium intercalary sclerites have been identified in the species of the Nepidae and Belostomatidae. A slightly different shape of intercalary sclerites and a significant reduction in their size (small) have been observed in two taxa (Ochteridae and Gelastocorinae). A similar appearance of medium-size subtriangular intercalary sclerites, however, wider in the Nerthrinae has been noted in two different taxa of the Nerthrinae and Aphelocheiridae. A curious example of differentiation of the shape and size of the intercalary sclerites has been noticed in the Naucoridae and Notonectidae. Both taxa are characterized by small sclerites, but the typical structure is present only in one subfamily, that is, that of the Cheirochelinae (Naucoridae). In two other subfamilies (Limnocorinae and Cryphocricinae), the basal membrane is slightly reduced in comparison to the previous groups. In this case, the plates of the sclerites are distinctly formed. In the remaining taxa of the Naucoridae (Laccocorinae and Naucorinae) and in all Notonectidae, the intercalary sclerites are vestigial; that is, only their distal part is slightly protruding. The boundaries separating the sclerites from the rest of the third labial segment can be distinguished by traces of a membrane. The presence/absence of the membrane (ms) at the base of sclerites can be a factor determining the categorization of these structures into well-developed ones characterized by the presence of the membrane (it is also present in the previously mentioned families) and poorly developed ones (the absence of the membrane (ms) can be viewed as a secondary reduction). 

The membranous separation of the intercalary sclerites from the rest of the labium apparently increases the efficiency of the holdfast mechanism and allows for a more independent movement of these sclerites, as has been noticed in the Nepidae, Belostomatidae, Ochteridae, Gelastocoridae, and Aphelocheiridae [20]. In these taxa, the holdfast mechanism (general description in Parsons [20, 25]) includes the arms of the Y-shaped muscular device embracing the stylet groove; these muscles are connected with a pair of intercalary sclerites, separated from the third segment by extensions of the intersegmental membrane. 

In description of the holdfast mechanism in the *Notonecta* and *Ambrysus,* it has been shown that the muscles embracing the stylet groove are only attached to the lips (margins) of the stylet groove of the third segment, but they are not connected with the intercalary sclerites. One can assume that in the remaining subfamilies of the Naucoridae and Notonectidae, the intercalary sclerites are reduced, so the holdfast mechanism is the same as in *Ambrysus* (Naucorinae) and *Notonecta* (Notonectidae) described by Parsons [20]; that is, there is no direct connection between the muscles and the sclerites.

A direct connection of the membrane of the internal sclerites with the muscles (holdfast mechanism) maintains a well-developed and efficient action of these sclerites, as has been presented in five systematic families that represent a common model. The subfamily of the Cheirochelinae, among five tested subfamilies of the Naucoridae has retained this model; however, the type of the holdfast mechanism is not known. The holdfast mechanism formed in this way probably influenced the reduction of the intercalary sclerites in the discussed taxa (Laccocorinae, Naucorinae, and Notonectidae). 

The study has demonstrated the presence and similarity of the intercalary sclerites in some of the nepomorphan species and especially in all studied naucorid species, leading to a new outlook on the evolution of these structures. According to Parsons [25], these structures have little if any taxonomic significance because they occur sporadically among the nepomorphans; however, they are present and well-developed in the Nepidae, Belostomatidae, Gelastocoridae, Ochteridae, and Aphelocheiridae, but, on the other hand, in some of the Naucoridae (*Ambrysus*,* Pelocoris*,* Limnocoris*), they are poorly developed or absent as in the *Cryphocricos* and * Heleocoris*; they are also absent in the Notonectidae. 

Moreover, Mahner [12] wondered whether the intercalary sclerites that occurred only in some Cryptocerata, and in the Gerromorpha, Pachynomidae, Plokiophilidae, and Reduviidae, formed a common basal pattern or whether they had been formed independently, several times, within the Heteroptera and in particular the Cryptocerata, where they have evolved at least three times.

Modification of the intercalary sclerites across all Nepomorpha seems to be clearly defined. The loss of the muscle connection with the intercalary sclerites has restricted their movement and the function of the membrane has lead to reduction of these sclerites in three taxa that are more advanced from the evolutionary point of view. It seems that these structures have evolved at least two times as well-developed sclerites and as vestigial sclerites. It is contrary to the theory proposed by Parsons [25], who reported that the intercalary sclerites apparently enabled the holdfast mechanism to operate more efficiently and could have evolved independently several times in the Hydrocorisae in response to functional needs. Presently, the data on the kind of the holdfast mechanism is insufficient with respect to the Pleidae and Helotrephidae, and (probably it is similar to the holdfast mechanism in other groups of Nepomorpha), so it is not possible to review this mechanism in relation to the loss of the sclerites in these families. However, Cobben [18] ambiguously underlined the presence of the semicircular reinforcement (= holdfast mechanism?) of the stylet groove in the helotrephid *Idiotrephes*. 

For the inspection of size and shape of the sclerites, the basal form can be estimated from the most plesiomorphic taxa, such as the Belostomatidae and Nepidae [9, 12, 17, 25, 37] in order to compare them to the intercalary sclerites of some gerrids. Cobben [18] summarized that the sclerites of gerrids had large lobes and were similar to the sclerites of some Nepomorpha, so theoretically sclerites of the belostomatids and nepids represented the plesiomorphic condition. Due to the previous assumption, the structure of the intercalary sclerites deviating from belostomatids and nepids has been regarded as a recent evolutionary development. 

Mahner [12], in his phylogenetic considerations coded the completely reduced intercalary sclerites as separate autapomorphies for the Cryphocricini and Laccocorinae, and the vestigial intercalary sclerites as a plesiomorphy in the remaining subfamilies of the Naucoridae. Moreover, the same researcher regarded the completely reduced intercalary sclerites as a synapomorphy for the Notonectidae, Pleidae, and Helotrephidae.

In light of the present data, for the purposes of phylogenetic information, those sclerites can be reassessed as an synapomorphic character in the Nepidae and Belostomatidae as well as in the Ochteridae and Gelastocorinae and autapomorphies for the Nerthrinae and Aphelocheiridae (Table 3). Within the Naucoridae, three apomorphic states might be assigned for the subfamilies as an autapomorphy for the Cheirochelinae, synapomorphy for the Cryphocricinae, and Limnocorinae and an uncertain synapomorphy for the Laccocorinae and Naucoridae, and the Notonectidae. A lack of these sclerites is characteristic for the Pleidae and Helotrephidae. Cobben [18] noted the absence of the sclerites also in *Coleopterocoris* (Potamocoridae). 

A distinct trend of differentiation in the shapes of intercalary sclerites has been observed in other heteroptera taxa such as the Gerromorpha (from a basal family to more advanced families, Cobben, [18]; Brożek in prep.). Moreover, diversified shapes of intercalary sclerites have been found in the Plokiophilidae (only in the Embiophila), in several of the Reduviidae and in some Pachynomidae [18]. This process of differentiation of intercalary sclerites in various heteropteran taxa is probably common, so these elements might be regarded as potential systematic characters.

### 4.4. A Comparison of the First Segment across the Nepomorpha

Generally, characteristics of the first labial segment are of a similar type (ring-shaped segment) across the Nepomorpha except for the Corixidae. The previous studies [20, 21, 25], as well as the present study, have confirmed that in certain families and subfamilies there occur slight differences in the shape of the first segment. A well-developed ring in the absence of a well-defined stylet groove has been observed only in the Belostomatidae and in some of the Nepidae. This character is a rather plesiomorphic condition in these taxa regarded as a basal stem of the Nepomorpha [9, 12, 20, 30]. However, in some of the Nepidae, the first segment is well-developed only on the ventral side, while dorsally it has the appearance of a small ring visible in the *Laccotrephes* and invisible in the remaining tested species.

According to Hamilton [27], Quadri [21], and Parsons [20], this segment is reduced dorsally and in the Ranatrinae it has not been evidenced at all. The data gathered for the present study and the data gathered by the aforementioned authors indicates the autapomorphical character state of this segment. Several pieces of evidence for the Belostomatidae and Nepidae pointing out to similar characters of the labium and other anatomical structures have been provided by Parsons [20, 25, 38], and they have served as basis for distinguishing the previous groups from the Gelastocoridae, Ochteridae, Aphelocheiridae, Naucoridae, and Notonectidae.

In the present study it has been estimated that the segment in question is ring-shaped and also well developed in the Ochteridae and Gelastocoridae (Gelastocorinae), similarly as in the Belostomatidae, with the one exception: the stylet groove is closed along the whole length of the segment. Such characters as the edges being in contact on the dorsal side and the stylet groove being closed can be estimated as synapomorphies for these taxa. Undoubtedly, in the Nerthrinae (Gelastocoridae), this segment represents a slight deviation through the presence of the lateral incisions, and it can be interpreted as a new autapomorphical character for this subfamily. The other example of a new character indicating the autapomorphical condition of this segment is the lack of the midventral condyle in the Ochteridae in contrast to the presence of this condyle in the remaining families (symplesiomorphy). This process (midventral condyle) has been observed also in the Gerromorpha (Brożek, in prep.). 

In the Aphelocheiridae, this segment shows a mixed character and has been estimated as the autapomorphy. On the one side, it is similar to the Ochteridae and Gelastocoridae, being a well-developed ring, and on the other side its similarity to the Naucoridae is based on the fact that the stylet groove is open. According to other studies [4, 25, 39], on the Aphelocheiridae, there are also other intermediate characters: ovoid flattened bodies are similar to the Naucoridae, while the long labium is similar to the Ochteridae. 

A further trend toward the modification of the first labial segment seems to have taken place in the Naucoridae. Parsons [20] has pointed out the reduction of the first segment in *Ambrysus*, which is considerably shorter dorsally than ventrally, and its style groove is open. Presently, a similar shape of this segment has been observed in all investigated naucorid species and provides the same characters; it is a probable synapomorphy for the Naucoridae. These characters are important from the point of view of future phylogenetic analysis, due to the monophyletic character of this family indicated by Hebsgaard et al. [30] and the lack of synapomorphy reported by Brożek [19].

Another example of further differentiation of the first segment can be found in the Pleidae and Helotrephidae. A new type of segment (two times narrower ventrally than dorsally) can be identified as a synapomorphy for these families. Probably, this segment is a modified form derived from the lineage of the wide and long segment of the Belostomatidae and Nepidae which appears in more reduced forms in the Aphelocheiridae, Naucoridae, Helotrephidae, and Pleidae. China [40] has pointed out that the shortness of the basal segment produces the flexibility necessary for these predatory insects. However, the insects with a long basal segment are also successful as predators. The first segment of the Notonectidae is well-developed morphologically and resembles its counterpart in the Belostomatidae, which rather suggests the reversal state.

### 4.5. A Comparison of the Second Segment across the Nepomorpha

The presence of the uniform (undivided) plate on the dorsal side in the second segment in the following four families: the Nepidae, Belostomatidae, Gelastocoridae, and Ochteridae seems to be a plesiomorphic condition (it is in accordance with a similar shape of the second segment in the Gerromorpha, according to Andersen [33], contrary to the divided plate (triangular plate) of this segment in the remaining taxa, which is an apomorphic condition for the Aphelocheiridae, Naucoridae, Notonectidae, Pleidae, and Helotrephidae. Although strong evidence has been obtained on the fact that the triangular plates essentially represent the same type, the area in question is more exposed (nodule) (Figures 28 and 29) in the Pleidae and Helotrephidae and this character can be regarded as a synapomorphy for these taxa. In turn, a strong convex plate on the dorsal side of the second segment as well as the lateral wing-plate has been found in *Limnocoris lutzi* (Figures 23(b) and 23(c)) (Limnocorinae) as the example of a modification and assessed as an autapomorphy. Moreover, in the present study, a specific shape of the triangular plate has been indicated; that is, the nodules on the distal part of this area are distinctly visible in *Notonecta glauca* (autapomorphic character) while in the remaining species of the Notonectidae the triangular plate is planar. On the other hand, these nodules are smaller in *Notonecta* than in the helotrephids and pleids. However, the presently reported modification of these elements in these species probably suggests the possibility of the independent development of the second segment.

A dozen or so species of the Naucoridae, Notonectidae, Pleidae, and Helotrephidae have been examined by Mahner [12], Parsons [20, 25], Esaki, and China [41, 42] (*Idiocoris lithophilus, Helotrephes bouvieri, *and* Tiphotrephes indicus*) and some individual species have been examined by China [43] (*Neotrephes usingeri*), Esaki and Miyamoto [44] (*Helotrephes formosanus*), and Poisson [45] (*Paralimnotrephes villiersi*). On the basis of the collected data, despite clear differences in the shape of the triangular plate known to Mahner [12] between two groups of families, the Aphelocheiridae, Naucoridae and Notonectidae on one hand and the Pleidae and Helotrephidae as well as the Potamocoridae on the other, the shape of the triangular plate has been generalized and coded as a synapomorphy. In consequence, the afore mentioned taxa have been incorporated into a common group of the Cibariopectinata, although other characteristics have also been taken into account. According to present data, this aspect (Cibariopectinata) of the phylogenetic reconstruction of Mahner [12] is not supported by the estimation of the character of the second segment in this paper, neither is it supported by the molecular data gathered by Hebsgaard et al. [30].

### 4.6. A Comparison of the Third and Fourth Segments across the Nepomorpha

Strong evidence has been obtained on the fact that that the third and fourth segments essentially have the same shapes (tubular and conical, resp.) across all Nepomorpha (except for the Corixidae). Although within the nepomorphans the shape of this segment is fairly constant, there are several specific variations regarding its length. Generally, in this infraorder, the third segment is longer than the other three. However, this is most evident in the Ochteridae, Nerthrinae, and Aphelocheiridae, in which the longest third segment is about six times longer than the others, while in the remaining families this segment is no more than two times longer in relation to other segments. With respect to the Ochteridae, Nerthrinae, and Aphelocheiridae, the evaluation of this character is ambiguous. It can be considered as a plesiomorphy, because there is no other evidence on the existence of a close relationship among these three taxa or it can be considered as a convergence character; the long III segment (long labium) has developed independently in these groups. The plesiomorphic character in the previously mentioned taxa can be evidenced with respect to the Gerromorpha where the third labial segment is by far the longest in relation to the remaining segments [33]. On the other hand, Mahner [12] has pointed out that the long III segment is a convergence character for the Ochteridae and Aphelocheiridae. 

However, other characters of the labium, as other parts of the body of the ochterids are resemble those of the closely related Gelastocoridae [39]. Another point of view on the labium of the gelastocorid has been proposed by Popov [9]; the Nerthrinae labium (III segment) is evidently longer than that in the Gelastocorinae. According to the data gathered by Popov, [9] the synapomorphy is more probable between the Ochteridae and Nerthrinae.

An evident modification, that is to say, a significant difference in the length of the labial segments, has been observed in the Helotrephidae (four species have been examined in the present study; see Figures 29(a), 29(c), and 29(g)), in which the fourth segment is dominant (being three to four times longer than the others). The elongated fourth segment of the labium in the two other species (*Paratrephes bintoni* and *Helotrephes bouvieri *(see Figures 17. Kb, Kc in Mahner's manuscript)) has been indicated by Mahner [12], and thus his data supplements and confirms the presently analyzed characters of the Helotrephidae. Undoubtedly, we have to do with a series of transformations leading towards the extension of the distal labial segment that represents the state of autapomorphy in this family. There is also evidence that in one helotrephid species, namely, *Neotrephes usingeri,* the fourth labial segment is as short as in the Pleidae. According to Mahner's [12] interpretation of this fact, this feature in the *Neothrephes usingeri* has been inherited from the ancestral species of the Pleidae (*Plea minutissima*, *Neoplea striola*, and *Paraplea frontalis*). 

Significant variations have also been identified with respect to the position and presence of the midvental condyle between the third and fourth segments. According to the data previously gathered by Parsons [20, 25], the midventral condyle is present in all Nepomorpha (Hydrocorisae) on the distal edge of the third segment. 

In this study, the presence of the condyle has been confirmed only in several taxa, such as the Belostomatidae (six species investigated), Nepidae (six species), and Cheirochelinae (three species) (Naucoridae). In such families as the Ochteridae, Gelastocoridae, and Aphelocheiridae, the condyle has not been observed (as documented by SEM images). Another example of modification has been found in the remaining taxa (Naucoridae except for the Cheirochelinae, Pleidae, Helotrephidae, and Notonectidae) where the midventral condyle has been formed on the proximal edge of the fourth segment.

Probably, the condyles at the third and fourth segments should not be treated as homologous structures. However, any discussion on the homologies of these condyles is presently premature because so far no exact data on this subject has been gathered regarding the other heteropteran groups. The results of this study only suggest the existence of evolutionary trends regarding changes of these condyles. The presence of the condyle on the third segment in the basal taxon of the Belostomatidae and Nepidae indicates their plesiomorphic condition and the reversal state in the Cheirochelinae. The other trend evidently represents the loss of this condyle in the Ochteridae, Gelastocoridae, and Aphelocheiridae that are arranged on the higher systematical level, which can be construed as their apomorphic character. In the following systematic taxa (Naucoridae except for the Cheirochelinae, Pleidae, Helotrephidae, and Notonectidae) it seems that the condyle has been formed “de novo” on the fourth segment and this character can be also perceived as a synapomorphy for these taxa.

Some remarks may be made upon condyle characters in relation to their function. However, they can only be speculative (due to lack of exact data). Nevertheless, it seems that the loss of flexibility of the intercalary sclerites can be connected with the appearance of the condyle on the fourth segment. Both cases have occurred in the same taxa (Naucoridae except for the Cheirochelinae, Pleidae, Helotrephidae, and Notonectidae).

Several other structures on the labium indicate the variation in the Nepomorpha and consequently four ways of articulation have been distinguished between the third and second segments on the dorsal side. Thus, a basal type is generally common in most taxa and is treated as a plesiomorphy. The other type has been indicated for the Ochteridae and it is viewed as an autapomorphy, similar as more complex articulation type in the Nerthrinae (autapomorphy). The resemblance of that connection in the Pleidae and Helotrephidae is regarded as a synapomorphic character. In a description provided by Parsons [20, 25], the articulation in this place of the labium has been recognized as one generalized type for all nepomorphan taxa.

A summary of the characters of the labial segments (I–IV) are displayed in Table 4. 

## 5. Phylogenetic Remarks and Conclusions

In relation to studies on the labium conducted by Parsons [20, 25] and Mahner [12], the current morphological characters of the labium have been compared among several species other than those studied in the works of those authors. Thus, the range of the examples has been wider and can serve the purpose of enriching the set of characters useful for further phylogenetic analysis. 

The present analysis of the set of characters has not led to proposing any new phylogenetic solution regarding these taxa but has rather been a survey aiming to determine whether the apomorphic character of the labium permits establishing some classifications and relationships with respect to these taxa in the groups that have already been recognized according to [9, 12, 20, 30, 31, 37]. These authors provide various hypotheses with respect to phylogenetic relationships among the nepomorphan taxa, so a pool of morphological characters can be helpful in reconsideration of these views.

The new apomorphic characters listed previously in the discussion section should be utilized in order to gain further insight into the phylogeny of the Nepomorpha. In the future a cladistic analysis should be conducted, taking into account selected characters and states determined for these taxa, possibly including also the analysis of the data that are presently missing (i.e., on the Corixoidea and Potamocoridae). However, several significant indications regarding the relationships among families of the Nepomorpha can already be pointed out based on the currently established characters (apomorphies).

1. Among most plesiomorphic characters of the labial segments of the Nepoidea (Nepidae and Belostomatidae), apparently there is only one character (palm-shaped apical plate of the last labial segment) viewed as a synapomorphy for the Belostomatinae and Lethocerinae; in the present study, it provides support for the monophyly of the Belostomatidae. Moreover, the longest second segment is the state of autapomorphy for the Belostomatinae. Furthermore, a weakly developed first segment on the dorsal side represents a synapomorphy for the Nepinae and Ranatrinae, which also points to the monophyly of the Nepidae. This result supports the monophyly of the Belostomatidae and Nepidae that has also been recognized previously by several authors [9, 12, 30, 31, 37]. The first five of the previously mentioned authors have proposed hypotheses that agree with the presently made assumption that the Belostomatidae and Nepidae (Nepoidea) should be placed as a sister basal group of the Nepomorpha, whereas Hua et al. [31] have placed the Corixidae in the basal line in this infraorder.
2. In this study, it has been estimated that the closed stylet groove on the first and second segments indicates one synapomorphy for the Ochteridae and Gelastocoridae and establishes a sister group relationship between them, supporting their presence at the superfamily level (Ochteroidea). The superfamily Ochteroidea [12, 30, 46] (Gelastocoroidea [9]) has been generally recognized in previous investigations. The four autapomorphic characters (the laterally the second segment is divided laterally, the apical plate has the shape of a slim palm, the wide subtriangular intercalary sclerites and the complex articulation of the third and second segments on the dorsal side) evidently point out the apomorphic character of the Nerthrinae and support the monophyly of this taxon. On the other hand, there occurs a synapomorphy (shape and size of the intercalary sclerites) for the Ochteridae and Gelastocorinae. Brożek [19] has recently noted substantial differences in the rupturing system and the length of the mandibular file between the Gelastocorinae and Nerthrinae (Gelastocoridae). According to Popov [9], the family Gelastocoridae is represented by two monotypic subfamilies and one of them, the Nerthrinae is closer to the Ochteridae and other ancestral families with the longer and thinner rostrum, which is slightly curved forward, similar to the Nerthrinae.
3. The monophyly of the Ochteridae has been supported by one autapomorphic character (the missing midventral condyle on the first segment on the ventral side).
4. The Aphelocheiridae have received one autapomorphy (sub-triangular intercalary overlapping the lateral side of the third segment).
5. The identical shape of the first segment presently observed in several species seems to be an indicator of the monophyly of the Naucoridae. Hebsgaard et al. [30] have classified the Naucoridae as monophyletic, whereas no synapomorphic characters of this family have been recognized by Brożek [19], who studied maxillae and mandibles. The remaining diversified characters of the labial segments are especially important within the Naucoridae. The shape of the apical plate has been diagnosed as a synapomorphy for four subfamilies apart from the Limnocorinae (reversal character). An autapomorphy has been diagnosed for the Cheirochelinae (small intercalary sclerites with the basal membrane) as well as a synapomorphy for the Cryphocricinae and Limnocorinae (intercalary sclerites without the basal membrane). The Laccocorinae and Naucorinae share the character of the vestigial intercalary sclerites with the Notonectidae.
6. The set of several characters (the extension nodules situated on the dorsal side of the second segment, the articulation between the second and third segments on the dorsal side) comprise two synapomorphies for the Pleidae and Helotrephidae and one autapomorphy for the Helotrephidae (the long fourth segment). The clade Pleidae + Helotrephidae (Pleoidea), pointed out in many previous studies [9, 30, 37], has been confirmed in the present study as well. However, the concept proposed by Mahner [12], namely, Notonectidae + Pleoidea (Pleidae and Helotrephidae), has not been supported.
7. In the present study, one synapomorphy (trapezoid shape of the apical plate) has been evidenced for the Anisopinae and Notonectinae and it points out the Notonectidae as a monophyletic group.

In the future, this set of characters will be utilized to investigate the cladogenesis, together with other mouthpart structures such as the recently studied mandibles and maxillae characters [19] and labium sensilla characters in the Nepomorpha [47], and also in order to link them with other states of morphological structures listed in Table 5 by Hebsgaard et al. [30].

## Figures and Tables

**Table 1:** A list of taxa whose labial segments have been studied. Specimens illustrated in this paper are marked with an asterisk on the list.

Families	Subfamilies	Species	Authors
Nepidae	Nepinae	*Curicta granulosa *	De Carlo 1951
		Borborophyes mayri*	Stål, 1871
		Laccotrephes japonensis*	(Scott, 1874)
		*Nepa cinerea *	Linnaeus, 1758
	Ranatrinae	Cercotmetus asiaticus*	Amyot & Serville, 1843
		Ranatra chinensis*	(Mayr, 1865)
Belostomatidae	Belostomatinae	Belostoma flumineum*	Say, 1832
		Deinostoma dilatatum*	(Say)
		*Appasus major *	(Esaki, 1934)
		Hydrocyrius colombiae*	Spinola, 1850
		Limnogeton fieberi*	Mayr, 1853
	Lethocerinae	Lethocerus deyrollei*	(Vuillefroy, 1864)
Ochteridae		*Ochterus marginatus *	(Latreille, 1804)
		Ochterus piliferus*	Kormilev, 1973
Gelastocoridae		Gelastocoris oculatus*	(Fabricius, 1798)
		Nerthra nepaeformis*	(Fabricius, 1798)
		Nerthra macrothorax*	(Montrouzier, 1855)
Aphelocheiridae		*Aphelocheirus variegatus *	Kiritschenko, 1925
		Aphelocheirus aestivalis*	(Fabricius, 1794)
Naucoridae	Cheirochelinae	Cheirochela feana*	Montandon, 1897
		Gestroiella limnocoroides*	Montandon, 1897
		Coptocatus oblongulus*	Montandon, 1909
		Coptocatus kinabalu*	Polhemus D. 1986
		Tanycricos longiceps*	La Rivers, 1971
	Laccocorinae	Laccocoris hoogstraali*	La Rivers, 1970
		Heleocoris humeralis*	Signoret, 1861
	Limnocorinae	Limnocoris lutzi*	La Rivers, 1957
	Cryphocricinae	Cryphocricos hungerfordi*	Usinger, 1947
		Ambrysus occidentalis*	La Rivers, 1951
	Naucorinae	Ilyocoris cimicoides*	(Linnaeus 1758)
		Pelocoris femoratus*	(Palisot de Beauvois 1820)
		Macrocoris rhantoides*	Bergroth,
		Naucoris maculatus*	Fabricius, 1798
		Neomacrocoris handlirschi*	(Montandon, 1909)
		Namtokocoris siamensis*	Sites 2007
Pleidae		Paraplea frontalis*	(Fieber, 1844)
Helotrephidae		*Helotrephes semiglobosus *	Stål, 1860
		*Hydrotrephes visayasensis *	Zettel, 2002
		Hydrotrephes balnearius*	(Bergroth, 1918)
		*Tiphotrephes indicus *	(Distant, 1910)
Notonectidae	Anisopinae	Anisops camaroonensis*	Signoret
		*Anisops sardea *	Herrich-Schäffer 1849
		Buenoa uhleri*	Truxal, 1953
	Notonectinae	Notonecta glauca*	Linnaeus 1758
		Enithares bergrothi*	Montandon, 1892
		Nychia sappho*	Kirkaldy, 1901

Specimens of the Potamocoridae were not available for the purpose of the present study.

**Table 2:** Morphology of the Labial Segments in the Nepomorphan Taxa.

*Belostomatidae (Belostoma flumineum and Lethocerus uhleri). *The* first labial segment* is much longer dorsally than ventrally. Its dorsal surface is indented, forming a very broad, open stylet groove, and the central, concave portion is separated from the lateral region of the segment by a narrow membrane. The stylet groove has no definite lips, the stylet bundle being held in place by the long, narrow labrum-epipharynx. The* second segment* is the longest of the four in the *Belostoma*, but it is shorter than the third segment in the *Lethocerus*. The intercalary sclerites between the third and fourth segments are larger than in the *Gelastocoris*, those of the *Lethocerus* being especially pronounced. Their length causes them to overlap the base of the *fourth segment*, to which they are attached with a long membrane. Both genera possess the apical plate.	*Nepidae *(*Nepa apiculata* and *Ranatra fusca*). *The first segment* is greatly shortened and is incomplete dorsally, being sclerotized only ventrally and laterally. *The second labial segment* is similar to that of the Belostoma. Proximally, it is constricted; it bears ventrolateral flanges and lacks a stylet groove; distally, it is wider, with a deep, closed stylet groove which is reinforced by the median process. In both genera, *the third labial segment* is the longest of the four. Intercalary, sclerites are present and are especially large in the Nepa. Both genera bear an apical plate in the stylet groove of the last segment.

*Gelastocoridae (Gelastocoris oculatus). The first labial segment* is quite long dorsally but much shorter ventrally, its latter portion being entirely concealed in the retracted labium, by the posterior plate of the cranium. In the first segment the sclerotized style groove is very deep and, unlike that of other Hydrocorisae, closed distally. The lips of the groove are separated only proximally, where they are overlapped by the short labrum-epipharynx. Ventrolaterally, the base of the second segment forms flanges articulating medially with the proximal segment, as in all other Hydrocorisae. *The second labial segment* is somewhat wider apically than basally. Its dorsal surface is not modified as that of the *Notonecta* and *Ambrysus*, and the lateral basal margins of the third segment do not project as far into its lumen as in those two genera. Two dorsolateral articulation points are present between these two segments. *The third segment* is the longest of the four, although proportionally shorter than that of the *Notonecta*. A pair of small intercalary sclerites (IS) flanks the stylet groove and is separated from the third and fourth segments by extensions of the intersegmental membrane. A similar arrangement is found in the Belostomatidae and Nepidae examined in this study, in the *Ochterus* (Ochteridae), and in *Gerris*. The nature of the sclerites from the remaining part of the labium indicates that they apparently increase the efficiency of the holdfast mechanism and allow it to move more independently. As for the *fourth labial* segment, proximally, its stylet groove is supported by a ventrally complete sclerotized ring (BS) which connects dorsally with the lips of the groove at the base of the segment.	*Aphelocheiridae *(*Aphelocheirus aestivalis*). *The first labial segment* is the broadest of the four; it is quite short and its greatest length occurring dorsolaterally and at the stylet groove. Its thickened ventral wall bears a pronounced median internal process which articulates with a similar process on the second segment. The dorsal wall of the first segment is thin and nearly transparent except at the stylet groove. Laterally, the distal margin of the first segment is thickened, this thickening perhaps representing the region which has given rise to the ventrolateral intersegmental sclerites in many Naucoridae. *The second labial segment* articulates with the first by means of a single midventral condyle which allows the segment a wide range of movement. In the second segment, the floor and sides of the stylet groove are membranous proximally. The membrane of the groove is attached proximally to a median marginal thickening of the ventral wall of the oblique plate. The exposed dorsal surface of the second segment is divided, by a narrow membrane, into proximal and distal portions. The distal portion consists of a pair of flattened, triangular sclerites flanking the stylet groove and extending laterally as far as the articulations between the second and third segments. The greatly elongated segment is dilated basally, but its distal two-thirds are more slender and tapered. The stylet groove is closed dorsally by sclerotized lips. Proximally, the floor of the groove is membranously connected to the median process, as in the second segment. Between *the third *and* fourth segments* the stylet groove is flanked by a pair of subtriangular intercalary sclerites which lie within the intersegmental membrane and form the lips of the stylet groove between the two segments.

*Naucoridae *(*Ambrysus magniceps*). *The first labial segments* are relatively broader and shorter. The first segment is reduced and, when the labium is swung posteriorly, entirely concealed within the head. Its style groove is open, sclerotized, and marginally thickened; a short membranous region placed distally, continuous laterally with the intersegmental membrane, joins it to the dorsal wall of the oblique plate. Laterally, the intersegmental membrane between the first two segments contains three intersegmental sclerites which lie very close together and often appear to be a single sclerite. Ventrolaterally, the base of the second segment is turned inwards, forming flanges which articulate midventrally with a process from the first segment as in the *Notonecta*. The dorsal surface *of the second segment* is divided into a proximal, concave portion and a distal flattened, triangular portion in all Naucoridae. This separation extends as far laterally as the dorsolateral articulation between the second and third segments. The stylet groove of this segment is membranous and closed dorsally; its floor is reinforced by a stout median process which extends distally into the third segment and is separated proximally from the oblique plate by a short membrane.	*Pleidae (Plea minutissima)*. In *the second segment* on the dorsal side, there is the triangular structure with the distal bead-like thickening [12]. Labium, short with a small apical labellum [4]. *Helotrephidae (Helotrephes semiglobosus)*. *The fourth segment* of the labium is the longest in most species except for the Neotrephes. *The second segment* a representative of likewise shows an anterior ridge-like thickening. *The second segment* is otherwise very narrow [12]. Labium, short with a small apical labellum[4]. *Notonectidae (Notonecta undulata). First labial segment*: it is well developed. The stylet groove is open and covered dorsally by the flap-like labrum-epipharynx. Laterally, the distal margins of this first segment are slightly sclerotized. *Second labial segment*: on the dorsal side, there is a flattened triangular area on both sides of the closed stylet groove. The apices of the two triangles are somewhat raised and they project proximally. Together, they form a lid over the stylet bundle at the base of the second segment. Laterally, the segment is somewhat swollen in the dorsal part. There is a dicondylic joint between the second and third segments; the articulation points occur dorsolaterally, at the lateral edges of the flattened triangular areas of

The terminal portion of the labium resembles that of the *Notonecta* except for the fact that the fourth segment is relatively shorter and broader and the sclerotized basal process reinforcing its stylet groove encircles the groove ventrally. The intercalary sclerites are poorly developed in most naucorids (*Ambrysus, Pelocoris, Limnocoris*), and are absent in some genera (*Cryphocricos* and *Heleocoris*).	the second segment. Lateral extensions of the base of the third segment project into the second segment, just ventrolaterally with respect to the articulations. *The third labial segment* is the longest of the four; it tapers distally and the membranous stylet groove is closed.

**Table 3:** A map of the characters of the apical plate and intercalary sclerites (marked with various colors) indicates the common or individual characters for particular taxa. SA: synapomorphy, AA: autapomorphy, PS: plesiomorphy, RW: reverse, CO: convergence.

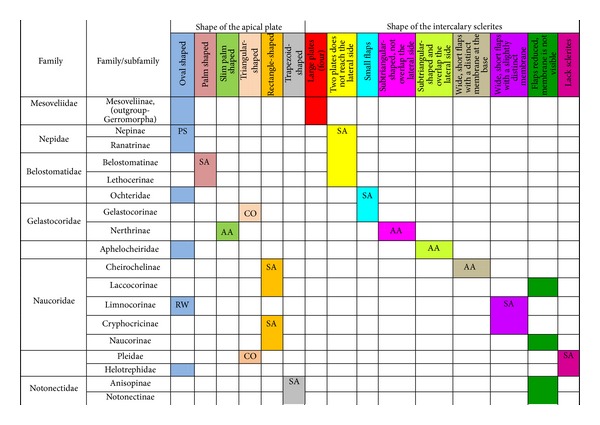

**Table 4:** Diversified characters of the labial segments (marked with various colors) are shown as common or individual characters for particular taxa. The characters indicated as “invisible” refer to unidentified characters (it was difficult to define these characters on the basis of SEM images). The table also includes characters of the outgroup (Gerromorpha) that are also analyzed in the Discussion.

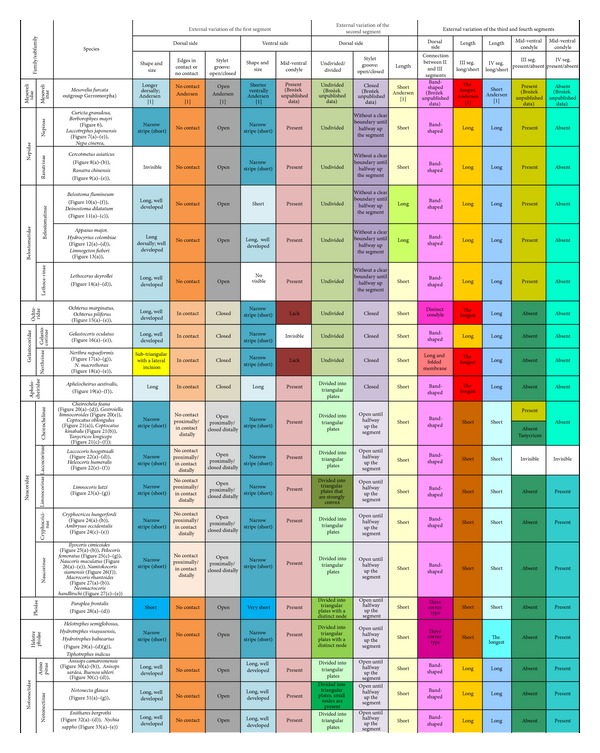

**Table 5:** The morphological data matrix for forty-one species of Nepomorpha and two outgroup taxa according to Hebsgaard et al. [30]. All sixty-five characters were given equal weight in the analyses and scored as binary and unordered.

	11111111112222222222333333333344444444445555555555666666
	12345678901234567890123456789012345678901234567890123456789012345
*Mesovelia furcata *	00000000000000000000000000000000000000000000000000000000000000000
*Saldus saltatoria *	10000000000000000000000000000000000000000000000000000000000000000
*Belostoma lutarium *	11111110000000000000000000100000000000010000000010000000000000010
*Lethocerus distinctifemur *	11111111000000000000000000100000000000010000000010000000000000010
*Lethocerus sp. *	11111111000000000000000000100000000000010000000010000000000000010
*Curicta scorpio *	11111000111100000000000000100000000000010000000010000000000000010
*Nepa cinerea *	11111000111110000000000000100000000000010000000010000000000000010
*Austronepa angusta *	11111000111111100000000000100000000000010000000010000000000000010
*Ranatra linearis *	11111000111111111000000000100000000000010000000010000000000000010
*Ranatra australis *	11111000111111111000000000100000000000010000000010000000000000010
*Gelastocoris oculatus *	11000000000000000111111111111111000000000000000000000000000000011
*Gelastocoris rotundatus *	11000000000000000111111111111111000000000000000000000000000000011
*Nerthra fuscipes *	11000000000000000111111111111100000000000000000000000010000000011
*Ochterus marginatus *	11000000000000000111111111000000110000000000000000000000000000011
*Ochterus sp. *	11000000000000000111111111000000110000000000000000000000000000011
*Diaprepocoris zelandiae *	11000000000000100110000000000000000011000000000000000000010000001
*Cymatia bonsdorffii *	11000000000000100110000000000000000011111111110000000000010000001
*Cymatia coleoptrata *	11000000000000100110000000000000000011111111110000000000010000001
*Agraptocorixa eurynome *	11000000000000100110000000000000000011111111001100000000010000001
*Glaenocorisa propinqua *	11000000000000100110000000000000000011111111001100000000010000001
*Corixa dentipes *	11000000000000100110000000000000000011111111001100000000010000001
*Potamocoris sp. *	11000000000000000111110000000000001100010000000000000000000000000
*Asthenocoris luzonensis *	11000000000000000111110000100000001100010000000011100000000000001
*Ambrysus cal. bohartorum *	11000000000000000111110000100000001100010000000011100000000000001
*Cryphocricos hungerfordi *	11000000000000000111110000100000001100010000000011100000000000000
*Heleocoris sp. *	11000000000000000111110000100000001100010000000011100000000000000
*Naucoris maculatus *	11000000000000000111110000100000001100010000000011110000000000001
*Ilyocoris cimicoides *	11000000000000000111110000100000001100010000000011110000000000001
*Macrocoris sp. *	11000000000000000111110000100000001100010000000011110000000000001
*Pelocoris sp. *	11000000000000000111110000100000001100010000000011110000000000001
*Aphelocheirus aestivalis *	11000000000000000111110000000000001100010000000010001000000000000
*Aphelocheirus venus *	11000000000000000111110000000000001100010000000010001000000000000
*Notonecta glauca *	11000000000000100111110000000000001100010000000000000111001000000
*Notonecta lunata *	11000000000000100111110000000000001100010000000000000111001000000
*Buenoa confusa *	11000000000000100111110000000000001100010000000000000111100000000
*Anisops sardea *	11000000000000100111110000000000001100010000000000000111110000000
*Anisops debilis *	11000000000000100111110000000000001100010000000000000111110000000
*Plea minutissima *	11000000000000000111110000000000001100010000000000000110000110000
*Neoplea striola *	11000000000000000111110000000000001100010000000000000110000111000
*Paraplea sp. *	11000000000000000111110000000000001100010000000000000110000111000
*Hydrotrephes sp. *	11000000000000000111110000000000001100010000000000000110000100101
*Idiotrephes asiaticus *	11000000000000000111110000000000001100010000000000000110000100101

## Appendix

Photographical documentation (SEM images) of the labial segments characters among the Nepomorpha subunits (Figures 6–33).

## Abbreviations

Ap: Apical plate
bu: Buccula
bs: Base of the apical plate
cd: Dorsal condyle
cv: Midventral condyle
cp: Convex plate
en: End of the apical plate
gl: Orifice of the maxillary gland
gr: Stylet groove
mb: Intersegmental membrane
in: Notch (incision)
is: Intercalary sclerites
Lr: Labrum
Mxp: Maxillary plates
no: Nodule
nd: Nodule (distinct visible)
Pp: Posterior plate of cranium
si: Intersegmental sclerites
sp: Suspensory plate
stb: Stylets bundle
tp: Triangular plate
wp: Winged plate
I: First segment
II: Second segment
III: Third segment
IV: Fourth segment
(D): Dorsal view
(L): Lateral view
(V): Ventral view.
